# Large parental differences in chromatin organization in pancreatic beta cell line explaining diabetes susceptibility effects

**DOI:** 10.1038/s41467-021-24635-2

**Published:** 2021-07-15

**Authors:** Xing Jian, Gary Felsenfeld

**Affiliations:** Laboratory of Molecular Biology, National Institute of Diabetes and Digestive and Kidney Diseases, National Institutes of Health, Bethesda, MD USA

**Keywords:** Imprinting, Type 2 diabetes

## Abstract

Previous GWAS studies identified non-coding loci with parent-of-origin-specific effects on Type 2 diabetes susceptibility. Here we report the molecular basis for one such locus near the *KRTAP5-6* gene on chromosome 11. We determine the pattern of long-range contacts between an enhancer in this locus and the human *INS* promoter 460 kb away, in the human pancreatic β-cell line, EndoC-βH1. 3C long range contact experiments distinguish contacts on the two sister chromosomes. Coupling with allele-specific SNPs allows construction of maps revealing marked differences in organization of the two sister chromosomes in the entire region between *KRTAP5-6* and *INS*. Further mapping distinguishes maternal and paternal alleles. This reveals a domain of parent-of-origin-specific chromatin structure extending in the telomeric direction from the *INS* locus. This suggests more generally that imprinted loci may extend their influence over gene expression beyond those loci through long range chromatin structure, resulting in parent-of-origin-biased expression patterns over great distances.

## Abstract (web summary)

A SNP distant from the human *insulin* (*INS*) gene near the *KRTAP5-6* gene confers increased susceptibility to type 2 diabetes when present on the paternal allele while decreased susceptibility when on the maternal allele. Here the authors show that long-range contacts between the *INS* locus and the *KRTAP5-6* gene locus distinguish paternal and maternal alleles.

## Introduction

**Fig. 1:**
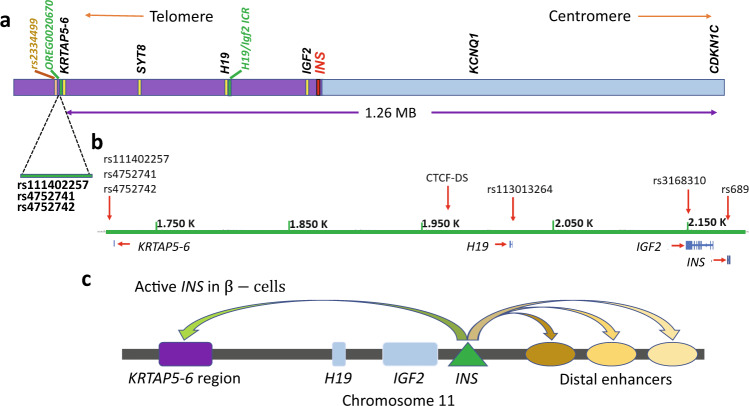
Features of human chromosome 11p15.5-p15.4 region. **a** Schematic representation of the key genetic elements in this study, ranging from 1.6 Mb to 3.0 Mb (hg19 assembly) from the telomere-end of chromosome 11. The purple and blue bars represent approximate extents of distinct TADs in this region (see Discussion). **b** The genetic features mentioned in this study, on human chromosome 11, from 1.7 Mb to 2.2 Mb (hg19 assembly). **c** The main 4C-seq contact pattern of the *INS* promoter in EndoC-βH1 cells. There is one major contact to the *KRTAP5-6* region telomeric from the *INS* gene, whereas there are multiple major contacts to distal putative enhancers centromeric to the *INS* gene.

The *insulin* (*INS*) locus on human chromosome 11 is embedded in a genomic region containing extended segments that are imprinted so that many of the surrounding genes are expressed in an allele-specific manner^1,2^. For example, in the *IGF2/H19* locus, *H19 imprinted maternally expressed transcript (H19)* non-coding RNA is expressed from the maternal allele, whereas the *insulin-like growth factor 2* (*IGF2)* gene is paternally expressed^3,4^. Toward the centromere end of this region, the potassium voltage-gated channel subfamily Q member 1 (*KCNQ1*) gene and the antisense *KCNQ1-OT1* long non-coding RNA are expressed from only one allele^5^ (Fig. 1a).

Human *INS* expression itself is not imprinted, except in the yolk sac^6^. However, sites at the telomere end of chromosome 11 nearly 500 kb away near the gene *keratin-associated protein 5-6 (KRTAP5-6)* (Fig. 1b and Supplementary Fig. 1a) have been shown to harbor type 2 diabetes (T2D) susceptibility loci that display parent-of-origin-specific effects^7^. This raises the possibility that an extended chromosomal domain might have direct or indirect effects on *INS* expression, and *INS* expression might in turn affect expression of distant genes^1,8,9^.

To explore this possibility, it is necessary to identify, in *INS*-expressing cells, long-range and potentially chromosome of parent-of-origin-specific physical interactions between elements in the regions surrounding the *INS* gene. We used the human pancreatic β-cell line, EndoC-βH1^10,11^, to focus initially on a region of chromosome 11 ~460 kb from *INS*, which has been reported^7^ to harbor a single nucleotide polymorphism (SNP), rs2334499, the T allele of which is associated with allele-specific effects on T2D susceptibility (Fig. 1a). This allele is linked with increased T2D incidence when paternally inherited but is protective when inherited maternally, indicating that some SNPs within the region confers an allele-specific phenotype affecting T2D susceptibility. In these cells rs2334499 is homozygous for the C (non-risk) allele (Supplementary Fig. 1b). SNP rs2334499 is located near the *KRTAP5-6* gene and it was suggested^7^ that rs2334499 might be associated with transcriptional factor CTCF (CCCTC-binding factor) binding in a nearby regulatory element, OREG0020670 (Fig. 1a), and which in turn might be responsible for allele-specific behavior. In previous publications^12–14^, this locus was referred as *MOB Kinase Activator 2 (MOB2) or HCCA2*. We find this nomenclature rather misleading, as in the current National Center for Biotechnology Information (NCBI) *Homo sapiens* Gene Annotation release 109.20210226 (https://www.ncbi.nlm.nih.gov/genome/annotation_euk/Homo_sapiens/109.20210226/) the coding region of *MOB2* gene has been reduced from previously 295 kb to 17 kb, so that rs2334499 and OREG0020670 is no longer within *MOB2* gene. The element OREG0020670 has been characterized in a previous genomic study in EndoC-βH1 cells, as shown in Supplementary Fig. 2^15^ (also available at https://shinyapps.jax.org/endoc-islet-multi-omics/). This element has the chromatin signature of an enhancer, and it is at the boundary between a euchromatic and a heterochromatic region.

Our earlier Chromosome Conformation Capture (3C) and Circularized Chromosome Conformation Capture (4C) measurements^1^ revealed strong contacts between the *INS* promoter and the *KRTAP5-6* locus in EndoC-βH1 cell line. For this study, we also re-analyzed our 4C data^1^ with a newer analysis method, 4C-ker^16^. As shown in Supplementary Fig. 3a and b, this is one of the strongest long-range interactions detected by our 4C data using the *INS* promoter as the bait sequence. When we decreased the k-value (the number of observed fragments to be analyzed within each window) from 10 to 3, we observed an increase of the strength of this contact. Meanwhile, the strength of most nearby contacts were decreased (Supplementary Fig. 3c). This new analysis is consistent with our previous analysis^1^. It is not known whether those long-range interactions occur on both chromosomes or preferentially on one, establishing a very long domain of allele-biased expression involving the *INS* gene indirectly or directly. Here we address this question by extended sequencing of the products of 3C experiments in EndoC-βH1 cells, coupled with identification of multiple allele-specific SNPs present in these cells. Combining these results allows us to identify a series of long-range contacts between the *H19/IGF2* imprinted control region (*H19*-ICR) and the *KRTAP5-6* locus, and sites between, that are confined in these cells to the maternal chromosome. We also map a contact between the *INS* promoter and *KRTAP5-6* that is largely specific to the paternal chromosome. These two sites appear to mark the 5’ and 3’ boundaries of a single topologically associated domain (TAD). We identify a CTCF site within OREG0020670 that is differentially methylated and occupied in a chromosome specific fashion, consistent with a parent-of-origin-specific contact pattern. Finally, we use a SNP in an *INS-IGF2* read-through transcript to show that in these cells *INS-IGF2* is predominately expressed from the paternal allele. Our results establish a network of contacts extending over 460 kb that physically couples the *INS* locus to distant regulatory elements in a parent-of-origin-specific fashion. In principle, this can result in unequal expression of *INS* from the two alleles.

## Results

### SNP rs2334499 and its properties

Genome-wide association studies (GWAS) have identified the SNP rs2334499 (T/C) as a T2D susceptibility locus in humans^7^. Allele T of rs2334499, at 11p15.5 (Supplementary Table 1), shows only a weak association [Odds Ratio (OR) = 1.08, *P* = 0.034] in the standard case-control test. However, when parental origin is considered, the paternally inherited allele (OR = 1.35, *P* = 4.7 × 10^−10^) makes a significant contribution to increased susceptibility. In contrast, the maternally inherited allele also shows significant association, but the effect of allele T is protective (OR = 0.86, *P* = 0.002). Kong et al. identified a regulatory element OREG0020670, about 17 kb centromeric to rs2334499 and 3 kb telomeric to the *KRTAP5-6* gene, as a 2 kb region with multiple variably methylated CpG residues. The authors showed that the T allele was correlated with decreased methylation of these sites, regardless of parental origin. They suggested that CTCF binding to sites also present in this region might be controlled by CpG methylation, thus coupling long-range chromatin organization by CTCF to rs2334499.

We began by exploring the corresponding region in the human pancreatic β-cell line, EndoC-βH1. Since OREG0020670 has been described as an enhancer in EndoC-βH1 cells (Supplementary Fig. 2)^15^, we decided to check whether deletion of the CTCF sites in this element would affect *INS* expression. We designed sgRNAs to target 3 CTCF motifs in this region (2 sgRNAs for each motif) by a recently developed CRISPR/Cas9 genome editing pipeline in EndoC-βH1 cells^17^. We quantified the editing events by a commercial T7-endonuclease I cleavage assay and found that editing most frequently occurred at the 5’- and middle-CTCF sites (24% and 27%), and the editing at the 3’-CTCF site was not efficient (6%) (Supplementary Fig. 4a, 5’-3’ orientation as Fig. 1b). As recommended by this protocol, we co-transduced multiple sgRNAs simultaneously into EndoC-βH1 cells, and we also detected large deletions between the 5’- and middle-CTCF sites by performing a PCR with primers on either side of the sgRNA target sites^17^ (Supplementary Fig. 4b). We compared *INS* mRNA levels from the cells after the CTCF-site-targeting sgRNA editing and cells from non-target sgRNA control and found that *INS* expression was significantly reduced, by 28% (Supplementary Fig. 5, *n* = 5, Student’s *t* test, *t* = 3.9599, *df* = 8, *p* = 0.0042, mean ΔΔCt = 0.47, 95% confidence interval of ΔΔCt: 0.20–0.74, 95% confidence interval of expression change: 0.60–0.87). As the current protocol cannot produce single clones of cells due to the difficult cell growth condition, we have not performed further functional studies on OREG0020670.

### *KRTAP5-6* and the *INS* locus

Although the *KRTAP5-6* locus (and OREG0020670) is about 460 kb distant from the *INS* promoter, our earlier 4C-seq results showed that in EndoC-βH1 cells there are strong contacts between these two sites on chromosome 11 (Fig. 1c and Supplementary Fig. 3)^1^. There are multiple other interactions between the *INS* promoter and sites in both directions along the chromosomes. Expression of many genes in this region is parent-of-origin-specific, and this raises the question whether the entire large domain from *KRTAP5-6* to sites centromeric to *INS* might be connected by a system of such specific contacts that could help control expression. We therefore made a series of measurements designed to determine whether the distant contacts identified by 4C-seq occurred equally on both alleles or biased toward one, and if the latter, whether in each case the contacts were preferentially on the maternal or paternal alleles.

We carried out 3C-PCR analyses to determine interactions among sites within the entire region between OREG0020670 and the *INS* promoter, taking advantage of the presence of three heterozygotic SNPs, [rs111402257(C/−C), rs4752741 (G/A), rs4752742(A/G); Fig. 1a/b, and Supplementary Fig. 6] to distinguish between contact patterns on the two alleles.

**Fig. 2:**
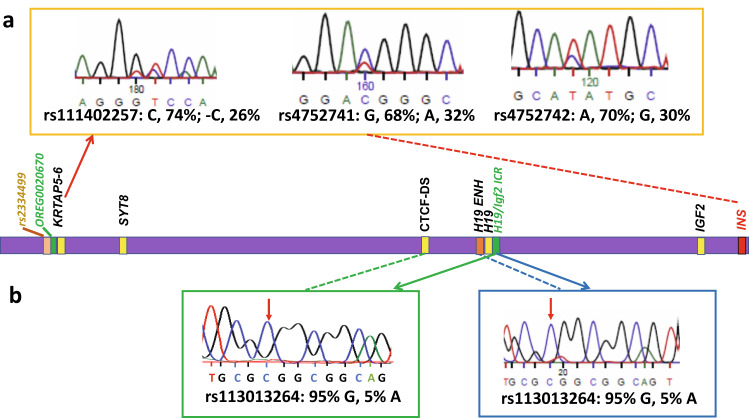
3C SNP analysis showing allele-specific chromatin contact patterns. **a** The contact between *INS* and OREG0020670 near the *KRTAP5-6* gene is enriched with allele rs111402257(C)/rs4752741(G)/rs4752742(A) over allele rs111402257(−C)/rs4752741(A)/rs4752742(G). The sequencing read is from the reverse strand. **b** The known mono-allelic contact pattern in the neighborhood of *H19* is conserved in EndoC-βH1 cells. Left: the contact between *H19*-ICR and CTCF-DS is enriched with the G allele of rs113013264 over A allele. The sequencing read is from the reverse strand. Right: the contact between *H19*-ICR and *H19* enhancer is enriched with G allele of rs113013264 over A allele. The sequencing read is from the reverse strand. Color coded arrows and dashed lines show contact patterns.

We first analyzed the products of 3C experiments using sites near *INS* and *KRTAP5-6* as anchors. The three SNPs in the *KRTAP5-6* region described above allowed us to distinguish alleles. Direct sequencing of the products revealed an ~2.5:1 bias in favor of the +C/G/A over the −C/A/G allele (Fig. 2a). This was confirmed by sequencing individual clones of the 3C products; 19 out of 21 clones contained the +C/G/A allele (Supplementary Fig. 7). On the other hand, PCR from a 1:1 mixture of synthetic 3C templates only showed slight amplification bias (~0.52:0.48) toward +C/G/A allele over −C/A/G allele (Supplementary Fig. 8). Thus, this long-range contact between *INS* and *KRTAP5-6* occurs preferentially on one of the two alleles. This observed bias in contact frequency cannot be attributed to the rs2334499 polymorphism because it is homozygous in EndoC-βH1 cells.

The question raised by these results is whether the specific contacts in the *KRTAP5-6* neighborhood are associated with the maternal or paternal allele. To determine this, we focused on the *IGF2/H19* locus about 300 kb centromeric to *KRTAP5-6*. Much of the underlying pattern of 3C contacts of this region has been described^18^ and the parent-of-origin-specific expression behavior is well documented^5,19,20^. *H19* is expressed primarily from the maternal allele and contacts between the *H19* enhancer and other relatively nearby sites are allele specific^18^. To map the contacts in this region we identified the (A/G) SNP rs113013264 (Fig. 2b), about 2 kb centromeric to *H19* transcription start site, located within the *H19*-ICR. Sequencing of 3C data anchored in the *H19* promoter and enhancer showed a strong bias for the G allele, identifying this allele as maternal (Fig. 2b). To support this assignment, we made use of the observation^18^ that the interaction between *H19*-ICR and a distal CTCF-binding site (CTCF-DS, ~50 kb away in the telomere direction) occurs mostly in the maternal allele in human B3 breast cancer cells. Examination of 3C contacts between these two sites (Fig. 2b) again shows that the G allele of rs113013264 preferentially contacts the CTCF-DS site (10/13G allele in cloned sequences, and >95% G over A allele in the 3C product), indicating this as the maternal allele (Fig. 2b and Supplementary Fig. 9). We also analyzed the methylation pattern of *H19-*ICR, as rs113013264 is located at one of the *CTCF*-binding site called hyper-sensitive site 5 (HS5). The A allele of rs113013264 would lack a CpG in HS5, which has been shown not affecting CTCF binding^21^, and is not conserved in other CTCF-binding sites at *H19*-ICR locus^19^. We obtained 17 clones of the G allele and detected fully unmethylated HS5 in all of them (Supplementary Fig. 10a). On the other hand, for the 4 clones of A allele obtained, we detected fully methylated HS5 (Supplementary Fig. 10b). This result indicates that *H19*-ICR has normal imprinted methylation pattern in EndoC-βH1 cells. It also confirmed again that G allele of rs113013264 is the maternal allele, and A allele is the paternal allele.

**Fig. 3:**
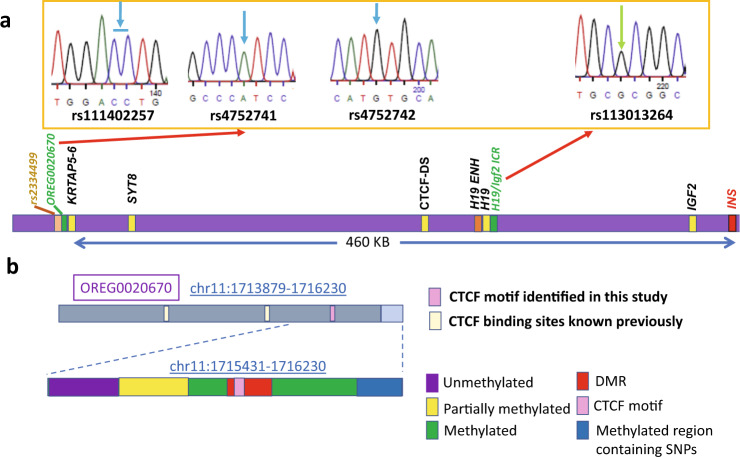
Assignment of the parental origin of the allele-specific contacts. **a** Sequencing read of a single clone of 3C-PCR product between *H19*-ICR and OREG0020670 near the *KRTAP5-6* gene showed that it contains G allele (marked with green arrow) of *H19*-ICR (rs113013264) and −C/A/G allele (marked with blue arrows) of OREG0020670 (rs111402257/rs4752741/rs4752742). The sequencing read is from the reverse strand for H19-ICR and from the forward strand for OREG0020670. Red arrows show contact sites. Sequencing results of eight additional clones of this 3C-PCR product is shown in Supplementary Fig. 11. **b** Key features of OREG0020670. Dark gray: euchromatin. Light grey: heterochromatin. Previously and newly identified CTCF sites^22^ are marked by small boxes. Enlarged bar graph represents the CpG methylation status in this region. Pink: CTCF motif (see Supplementary Figs. 6 and 14). Blue: methylated region containing SNPs rs111402257, rs4752741 and rs4752742. Purple: unmethylated region. Yellow: Variable methylated region without strong allelic specificity. Red: Differentially methylated region with allelic specificity. Green: methylated region. The DNA sequence (hg19 chromosome 11: 1715431–1716230) is shown in detail in Supplementary Fig. 6.

With this information we were able to determine the contact pattern of the *KRTAP5-6* locus. Making use again of rs113013264 we analyzed 3C products anchored in the neighborhoods of the *H19*-ICR and sites containing the three SNPs (rs111402257, rs4752741, rs4752742) in the *KRTAP5-6* locus. All (9 out of 9) obtained 3C products contained both the G allele near the *H19-*ICR and the –C/A/G allele in the *KRTAP5-6* locus (Fig. 3a and Supplementary Fig. 11), establishing that the −C/A/G allele, and this contact, are maternal. To confirm this assignment, we also analyzed two 3C products containing SNP rs113013264 at the *H19-*ICR but without SNPs at the *KRTAP5-6* locus (Supplementary Fig. 12). Both products also contained predominately the G allele at the *H19-*ICR (10 out of 10 cloned sequences, >99% G over A allele in the first 3C product, and >95% G over A allele in the second 3C product, Supplementary Figs. 12 and 13). We think this bias is due to the methylation of the paternal *H19*-ICR, which prevents CTCF-binding. This can be contrasted with the interaction determined above between the *INS* promoter and *KRTAP5-6* regions (Fig. 2a), which these results show to be specific principally to the paternal allele (C/G/A allele) at *KRTAP5-6* locus.

### CpG methylation patterns at OREG0020670

**Fig. 4:**
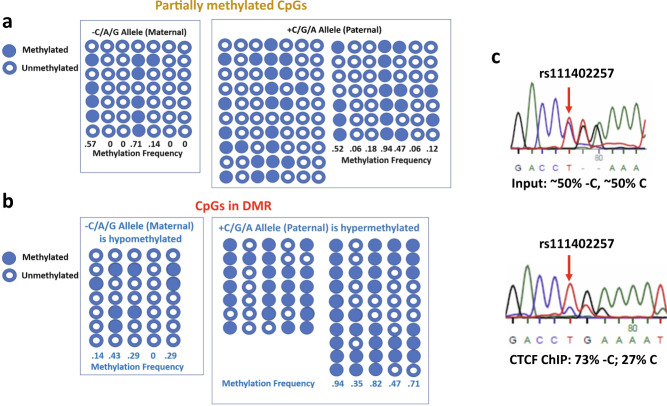
OREG0020670 shows allele-specific methylation and CTCF occupancy. **a** The seven CpGs in the variable methylated region (orange-colored CpGs in Supplementary Fig. 6) are partially methylated without strong allelic preference. **b** The CpGs in differentially methylated region (red-colored CpGs in Supplementary Fig. 6) are partially methylated with strong preference in the paternal allele, except for the second CpG. **c**. ChIP-PCR shows that CTCF preferentially binds to the maternal allele (−C allele of rs111402257). Top: genomic input sample. Bottom: CTCF-ChIP sample.

We carried out sequencing and bisulfite analysis of the region *chr11:1715431–1716230* (Fig. 3b), at the centromeric end of OREG0020670, taking advantage of the presence of three SNPs mentioned above, [rs111402257 (C/−C), rs4752741 (G/A), rs4752742 (A/G); Fig. 1] to distinguish between methylation patterns on the two alleles. These patterns (Fig. 4, Supplementary Fig. 6) vary: At the 5’ end of the sequence the CpG residues are unmethylated, but, in the region immediately downstream, methylation is partial, and randomly distributed between alleles, except for a single site that is highly methylated on both alleles (Fig. 4a, orange-colored CpGs in Supplementary Fig. 6). Beyond this we find a region extending for at least 500 bp within which levels of CpG methylation are high. Within this region, we find a CpG island showing an allele-specific methylation pattern, indicating a differentially methylated region (DMR): the +C/G/A allele is extensively methylated at a few CpG sites, whereas the −C/A/G is methylated at much lower levels (Fig. 4b, red-colored CpGs in Supplementary Fig. 6). we analyzed the sequence of this DMR with CTCFBSDB 2.0 (https://insulatordb.uthsc.edu/)^22^, and identified a CTCF-binding motif inside (Fig. 4c, Supplementary Figs. 6 and 14)^23^. Chromatin immunoprecipitation followed by PCR (ChIP-PCR) in EndoC-βH1 cells showed that CTCF preferentially binds to this site in vivo on the chromosome carrying the −C/A/G allele, which is the hypomethylated allele, and as shown above is the maternal allele. The binding motif for this CTCF site is immediately adjacent to, but does not overlap with, a methylatable CpG (Supplementary Fig. 6). We found that the CTCF zinc finger domain (containing all the eleven zinc fingers) could bind both the methylated and the unmethylated double-strand DNA (dsDNA) sequence in vitro (Supplementary Fig. 15), which is not surprising, as the CpGs are not within the CTCF-binding motif (Supplementary Fig. 6). The observed allele specificity of binding may not depend directly upon the methylation status of this site, and it may be connected to the chromatin structure of the entire region (see Discussion).

### Paternally biased expression of *INS-IGF2* transcript

**Fig. 5:**
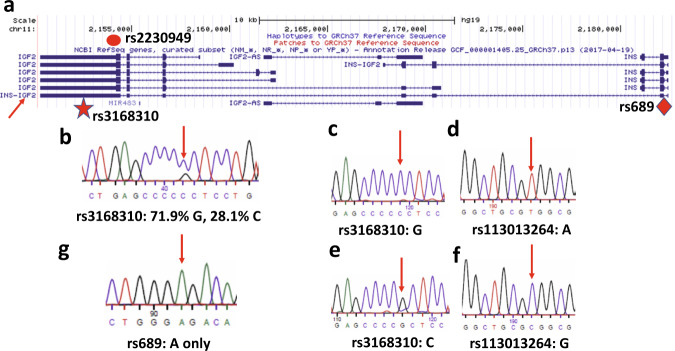
Paternally biased expression of *INS-IGF2* transcript. **a** Gene isoforms at *IGF2-INS* locus. Data is retrieved from UCSC Genome Browser^42^ hg19 assembly from chr11:2150251-2182750. Approximate position of SNP rs3168310 is marked with a star in the last exon of the *IGF2* gene, and that of SNP rs689 with a diamond in the first intron of the *INS* gene. Isoform 1 of *INS-IGF2* transcript is marked with an arrow. **b** Sequencing *INS-IGF2* isoform 1 PCR product shows that at SNP rs3168310, the G allele is the major product over C allele (71.9% G, 28.1% C). Sequencing primer reads from the reverse strand. **c**–**f** SNP typing *H19*-ICR-*IGF2* 3C product. The *IGF2* rs3168310 G allele (**c**) is found in the same PCR product with the *H19*-ICR rs113013264 A allele (**d**). Meanwhile, the *IGF2* rs3168310 C allele (**e**) is found in the same PCR product with the *H19*-ICR rs113013264 G allele (**f**). **g** Genotyping of rs689. Sanger sequencing showed that in EndoC-βH1 cells, SNP rs689 is the monoallelic A allele. The sequencing primer reads from the forward strand.

We hypothesized that the difference in chromosome contact pattern between the two parental alleles might affect the transcription activity of the *INS* promoter. The *INS* gene is homozygotic in EndoC-βH1 cells, so we could not determine whether there is any difference in the *INS* mRNA abundance between the two parental alleles. Since EndoC-βH1 cells only grow in high cell density, and form islet-like organoids, we were unable to introduce heterozygotic SNPs in the *INS* gene in an allele-specific manner by a conventional single-colony screening method. Instead, we decided to measure the *INS* promoter activity from an *INS-IGF2* read-through transcript. When the *INS* gene is transcribed in human cells, a small fraction of the mRNA fails to terminate at the 3’-end of the gene. Instead, the transcript is extended into the downstream *IGF2* gene (Fig. 5a). This transcript is expressed in a highly tissue-specific pattern: only in the pancreas is it expressed bi-allelically^24^. *INS-IGF2* was reported to be up-regulated in insulinomas^25^. There are two mature isoforms produced from this *INS-IGF2* read-through transcript, and it is still under debate whether the isoform 2 is involved in autoimmunity of type 1 diabetes (T1D)^26–29^.

The isoform 1 terminates at the 3’-end of the *IGF2* gene and is formed by splicing between the second exon of *INS* and the first exon of *IGF2*. We found that this isoform contains a heterozygotic SNP rs3168310 (G/C) at the last exon (Supplementary Fig. 16). We were able to amplify a fragment of this isoform by RT-PCR from the total RNA of EndoC-βH1 cells. The PCR primers are located within the 2nd exon of *INS* and the last exon of *IGF2*, so that only *INS-IGF2* read-through transcript would be amplified. Sequencing this PCR product showed that at SNP rs3168310, the G allele is the major product (71.9%) relative to the C allele (28.1%) (Fig. 5b). Thus, the isoform 1 of *INS-IGF2* is preferentially expressed from the G allele in EndoC-βH1 cells.

In the next step, we determined the parental origin for the G/C allele at SNP rs3168310. We successfully obtained 3C-PCR product that contains both rs113013264 in *H19*-ICR and rs3168310 in *IGF2* (Fig. 5c–f). By sequencing individual clones of this product, we found that the *IGF2* rs3168310 G allele is in the same PCR product with the *H19*-ICR rs113013264 A allele (Fig. 5c, d), and the *IGF2* rs3168310 C allele is in the same PCR product with the *H19*-ICR rs113013264 G allele (Fig. 5e, f). Since we already know that for rs113013264, A allele is paternal and G allele is maternal, we assign the G allele for rs3168310 to be paternal, and the C allele to be maternal. This assignment also agrees with the assumption that *IGF2* is predominately expressed from the paternal allele, as we obtain 5 times more clones of the 3C-PCR product from the assigned paternal allele than from the maternal allele. This assignment indicates that the isoform 1 of *INS-IGF2* is preferentially expressed from the paternal allele relative to the maternal allele. It is known that the *variable number tandem repeat (VNTR)* minisatellite polymorphism at the *INS* promoter would affect the expression of *INS* mRNA level^30^. This *VNTR* consensus sequence contains a binding site for transcription factor Myc-associated zinc finger protein (MAZ)^31,32^, which was recently reported to interact with CTCF^33^. We genotyped the nearby SNP rs689 which is in tight linkage disequilibrium with the *INS VNTR* (>99% in the Caucasian population)^34^. The sequencing results showed that rs689 is the homozygotic A allele (Fig. 5g), which indicates that in both the *INS* promoters, the *INS VNTR* class I allele is present. Thus, the large allelic bias we found in the *INS-IGF2* transcript level is not due to the *INS VNTR* polymorphism.

**Fig. 6:**
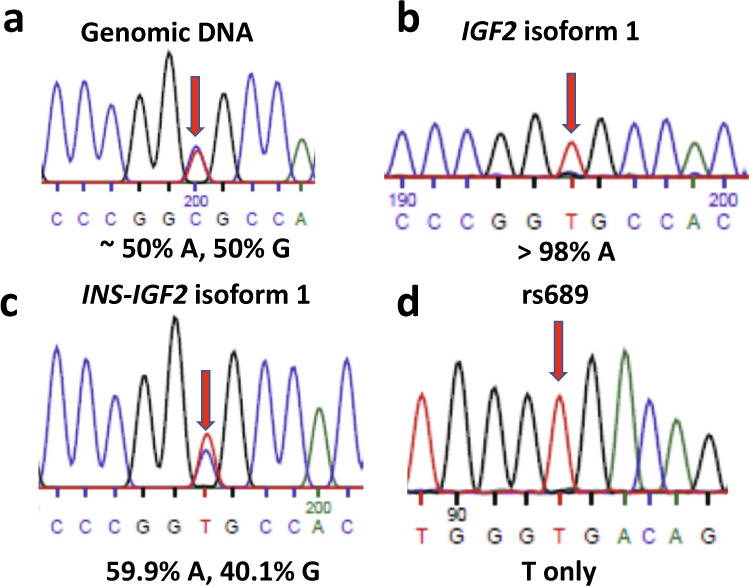
Allelic biased expression of *IGF2* and *INS-IGF2* in a human pancreatic islet sample. The sequencing primer reads from the reverse strand in **a**–**c**, and from the forward strand in **d**. a Genotyping of rs2230949 from genomic DNA, locating inside the last exon of *IGF2* gene. The locus is a G/A heterozygote. **b** Genotyping of rs2230949 of *IGF2* isoform 1 transcript. Only the A allele is expressed, indicating that this is the paternal allele. **c** Genotyping of rs2230949 of *INS-IGF2* isoform 1 transcript. The expression is biased toward the A allele. **d** Genotyping of rs689, showing that rs689 is the monoallelic T allele.

We obtained a human pancreatic islet sample to test whether paternally biased expression of *INS-IGF2* would occur in mature β-cells. The genotyping from genomic DNA showed that rs2230949 is a heterozygotic G/A locus in this sample (Fig. 6a). The sequencing result of *IGF2* isoform 1 transcript from RT-PCR showed that only the A allele of rs2230949 is expressed, indicating that this allele is the paternal allele (Fig. 6b). When we sequenced the *INS-IGF2* isoform 1, we found that the A allele is preferentially expressed (59.8 ± 1.9 %, from 3 portions of the sample, Fig. 6c, Supplementary Fig. 17). The sequencing results showed that rs689 is the homozygotic T allele in this sample (Fig. 6d), indicating that the *INS VNTR* class III allele is present in both *INS* promoters. In this human sample, it seems *INS-IGF2* is expressed preferentially from the paternal allele, matching with our observation in EndoC-βH1 cells.

## Discussion

By combining identification of allele-specific SNPs with extended sequencing of the products of 3C experiments, we have identified a large domain in a human pancreatic β-cell line containing the *INS* gene, in which chromatin organization is allele specific. This domain encompasses the *KRTAP5-6* locus at one end and the *INS* locus at the other, a distance of ~460 kb. The *KRTAP5-6* gene encodes a keratin-associated protein as a component of hair matrix^35,36^, which has not been reported to be related to diabetes. There is also no information about whether the SNP rs2334499 would affect its expression. This gene is expressed mainly in liver, placenta and skin, and has not been detected in pancreas^37^ (https://www.proteinatlas.org/ENSG00000205864-KRTAP5-6/tissue). Consistent with the presence of a domain boundary telomeric to *KRTAP5-6*, GWAS analysis has shown that *dual specificity phosphatase 8* (*DUSP8)* and *MOB2*, respectively ~140 and 220 kb telomeric to *KRTAP5-6*, are expressed biallelically^7^. We note that our results correspond rather closely with the extent of a TAD detected by Hi-C measurements (Supplementary Fig. 18) in EndoC-βH1 cells^15,38^. Any analysis of gene regulation must consider the fact that the maternal and paternal chromosomes could have quite different interaction patterns over the entire domain.

This study began with an examination of the SNP rs2334499, strikingly associated with T2D susceptibility in an parent-of-origin-specific manner. Our results strongly suggest that this behavior is connected to significant differences in chromatin organization between maternal and paternal chromosomes extending from rs2334499 to the *INS* gene. The differences we observe in EndoC-βH1 cells are not directly coupled to rs2334499 itself, as in these cells this SNP is homozygous. However, it seems likely that some SNP associated with the T allele of rs2334499 interacts with the extended specific domain structure that we describe, perhaps to affect *INS* expression in a parent-of-origin-specific manner. The results presented here establish remarkable, complex and distinct higher structural organizations for the two alleles. The complex series of interactions connects the entire 460 kb region between *KRTAP5-6* and *INS*, and strongly supports the idea that this is an extended locus with one set of interactions for the maternal and the other for the paternal allele. Although imprinting is observed centromeric to *INS* at sites such as *KCNQ1*, it seems likely that *KRTAP5-6/INS* constitutes a separately regulated domain.

Parent-of-origin-specific expression is typically associated directly or indirectly with parent-of-origin-specific DNA methylation^39^. Methylation of cytosine at certain CpG residues within a CTCF-binding site can disrupt its binding^19^. Because CTCF, through its recruitment of the cohesin complex, is well documented as helping to establish long-range contacts and ‘loop’ formation within the nucleus^40^, we looked for CTCF sites near *KRTAP5-6* that might contribute to domain structure in the observed specific manner. A partially methylated region with seven CpGs, shown by Kong et al.^7^ to display a methylation pattern correlated with the risk variant of rs2334499, did not show allele-specific methylation patterns in EndoC-βH1 cells (Fig. 4a, orange-colored CpGs in Supplementary Fig. 6), which do not carry that variant. However, in agreement with their observations, we find that the fourth CpG is most highly methylated; in EndoC-βH1 cells methylation is nearly complete on both alleles (Fig. 4a). It should be noted that Kong et al. observed that the parental origin would not affect the association between rs2334499 and methylation levels of these CpGs.

Because those CTCF sites cannot be related to the observed parent-of-origin-specific chromatin organization of *KRTAP5-6/INS*, we looked for other sites in the neighborhood and found a previously unrecognized CTCF-binding site (Supplementary Figs. 6 and 14). ChIP-PCR analysis (Fig. 4c) shows that this site is occupied by CTCF largely on the maternal allele, making it a plausible candidate for helping to organize *KRTAP5-6/INS* in a specific manner. This site is within a CpG island that is preferentially methylated on the paternal allele (Fig. 4b, red-colored CpGs in Supplementary Fig. 6), but the closest CpG is not at a location in the CTCF-binding motif that has been shown to disrupt CTCF-binding when methylated. Consistent with our methylation analysis, in a recently published dataset (http://meltonlab.rc.fas.harvard.edu/data/pancreatic_enhancers/)^41^, these five CpG sites showed partial methylation pattern in mature β-cells (methylation level: 0.5, 0.286, 0.636, 0.538, 0.417), although the allelic preference was not reported (Supplementary Fig. 19^42^). The average methylation level (0.475) is close to our results in EndoC-βH1 cells (0.444, Fig. 4b). Other cell types related to islet maturation process have higher methylation level. For example, in human pluripotent stem cells (hPSC), the methylation levels are 0.833, 1, 0.867, 0.955, 0.906, and in pancreatic α-cells, the levels are 1, 0.667, 0.9, 0.444, 0.636. Our current study is limited to EndoC-βH1 cells due to limited resource and time. In future studies, it would be interesting to investigate whether the methylation pattern in normal primary pancreatic β-cells is consistent with that found in EndoC-βH1 cells, and whether the samples from diabetic patients would show a different pattern.

In our in vitro assay, we did not detect any effect of DMR CpG methylation on the affinity between CTCF and dsDNA (Supplementary Fig. 15). We suggest that the low level of CTCF binding to the paternal allele might reflect its high extended level of CpG methylation in this region (Fig. 4b), known to be associated with heterochromatin formation^43^ and therefore of reduced accessibility to transcription factors. This is supported by Lawlor et al.^15^ who assigned this CTCF site to the edge of a 37.2 kb “weak repressed Polycomb” heterochromatin region centromeric to OREG0020670. Compaction of chromatin, independent of CTCF binding, might contribute to parent-of-origin-specific transcriptional behavior.

We observed that the histone 3 lysine 27 acetylation (H3K27ac) mark is enriched on the maternal allele (rs111402257 −C allele), the allele to which CTCF preferentially binds. On the other hand, we did not observe significant allelic bias of histone 3 lysine 9 trimethylation (H3K9me3) mark enrichment in this region (Supplementary Fig. 20). It is possible that another transcription factor binding site nearby would affect CTCF binding, and this transcription factor could be involved in DNA methylation. To aid future studies, we list the potential transcription factors that can bind to this region in Supplementary Table 2^44^.

**Fig. 7:**
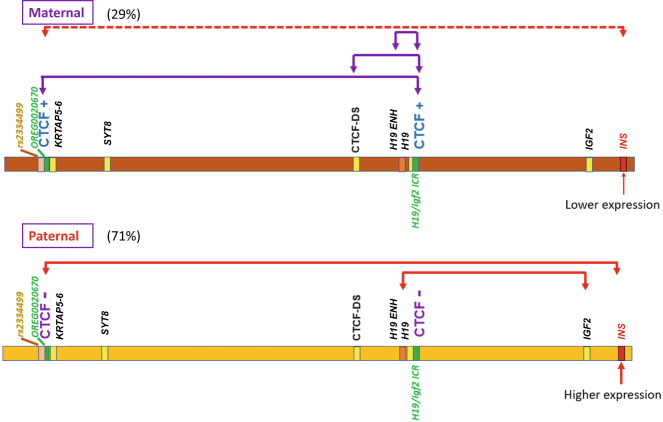
Summary of the parent-of-origin-specific contact patterns in the region between *INS* and OREG0020670 on chromosome 11. The contacts are indicated above the chromosome. The binding preferences of CTCF sites are also indicated.

We have identified a topologically discrete domain extending from *KRTAP5-6* to *INS*, within which the two chromosomes have quite different interaction patterns, probably coupled to distinct DNA methylation patterns. One of these interactions, restricted to the paternal allele, directly connects the *INS* promoter to the *KRTAP5-6* locus at the other end of the domain. Other interactions on the maternal allele connect *KRTAP5-6* with *H19* and the *H19-*ICR. This strongly supports the idea that the entire region is a single domain dominated by an imprinting mechanism. Parent-of-origin-specific contacts between the *H19* locus and *IGF2* have been described in earlier studies in mouse cells^45–47^ and are connected with parent-of-origin-specific expression of *H19* and *IGF2*. *KRTAP5-6/INS* is the larger domain which in humans encompasses all these contacts and differential expression patterns. Our results are summarized in Fig. 7.

The SNP rs2334499 is located at the telomeric end of *KRTAP5-6/INS*. The unusual property of the T allele, conferring opposite, parent-of-origin-specific T2D effects, was an important focus of the paper by Kong et al.^7^. The results we present here indicate that this SNP is located at the telomeric end of this large domain, and strongly suggests that within this domain there exist other SNPs that may confer allele and parent-of-origin-specific regulatory properties on expression of *INS* or other genes within the domain, such as *SYT8*^8^. As in the case of rs2334499, the contributions of these SNPs to T2D susceptibility may not be detectible without considering the parental origin. Further GWAS studies with extended populations may reveal such SNPs.

Our observation that the three dimensional organization of the entire region between *INS* and *KRTAP5-6* is parent-of-origin-specific suggests that there would be parent-of-origin-specific effects on *INS* expression, an idea consistent with the parent-of-origin-specific effects on T2D susceptibility associated with SNP rs2334499^7^. It has been accepted for many years that *INS* expression, unlike expression of the nearby *IGF2* and *H19* genes, is not imprinted except in the yolk sac. However, it was proposed in 1996 that the parent-of-origin effect of T1D susceptibility at *INS VNTR* locus might be mediated by an imprinted domain outside *INS VNTR*^30^. Because the *INS* gene does not contain heterozygotic SNPs in EndoC-βH1 cells, we identified SNPs in read-through extended *INS-IGF2* transcripts as a way of measuring allele specificity of expression of *INS* itself. These transcripts show a clear bias in favor of expression from the paternal *INS* gene. We were able to verify this result in a human sample, but in order to draw a general conclusion about the expression pattern of *INS* in the human population, many more samples must be tested. The *INS* promoter makes an unusual large number of genomic contacts (Supplementary Fig. 3a), and in this study we only analyzed parent-of-origin preference of one strong contact. While this result would indicate that the *INS* promoter in the two alleles are in different chemical environment, it would be prudent to not link parent-of-origin expression bias solely to the contact to *KRTAP5-6* locus. Only after examining other strong contacts as well, we can get a better understanding of how gene imprinting mechanism can affect *INS* expression. In addition, the interpretation of these results depends on the assumption that the abundance of the *INS-IGF2* read-through transcript is determined by the rate of transcription initiation at the *INS* promoter, and not by differences in rates of RNA splicing at a later stage, particularly important because the *IGF2* gene itself is not expressed from the maternal allele^48^.

Our observations provide a tentative explanation of how sites at the telomere end of chromosome 11 near the gene *KRTAP5-6* (Fig. 1a), which initially attracted our attention, can contain T2D diabetes susceptibility loci that display parent-of-origin-specific effects^7^ through interaction with large scale chromatin structure that differs dramatically on the two chromosomes. Thus, an imprinted locus can indirectly affect gene expression, recruiting distant regulatory elements in a parent-of-origin-specific manner. This may result in biased expression from one of the two chromosomes, as suggested above for the *INS* gene. An important conclusion of our study is that, because of the structural asymmetry involving contacts between *INS* and distant upstream elements, SNPs that affect *INS* expression should frequently have an overall effect that is parent-of-origin-specific. It seems likely that other imprinted loci will also help organize parent-of-origin-specific chromatin contacts for a considerable distance around them, and that this will have significant regulatory implications throughout the genome. Hi-C or other studies that do not distinguish contributions from the individual alleles may overlook important differences in chromatin structure and regulation of gene expression.

## Methods

### Culture of EndoC-βH1 cells

EndoC-βH1 cell line was obtained via a material transfer agreement between Inserm Transfert, CNRS, Endocells, and National Institutes of Health. EndoC-βH1 cells were cultured in low-glucose Dulbecco’s modified Eagle’s medium (Life Technology, catalog no. 11885) with 2% bovine serum albumin (BSA, fatty acid free and heat shocked, Equitech, catalog no. BAH66), 50 µM 2-mercaptoethanol, 10 mM nicotinamide (Calbiochem, catalog no. 481907), 5.5 µg/ml human transferrin (Sigma–Aldrich, catalog no. T8158), 6.7 ng/ml sodium selenite (Sigma-Aldrich, catalog no. S9133), 100 U/ml penicillin, and 0.1 mg/ml streptomycin. Cells were seeded at a density of 6.2 × 10^5^/cm^2^ on ECM-gel (1%) (Sigma-Aldrich, catalog no. E1270) /fibronectin (2 µg/ml; Sigma–Aldrich, catalog no. F1141) coated plates and cultured at 37 °C in a 5% CO_2_ incubator. The genomic coordination used in our experiments is based on Genome Reference Consortium Human Build 37 (GRCh37/hg19).

### CRISPR/Cas9 genome editing in EndoC-βH1 cells

The procedure to perform genome editing of OREG0020670 in EndoC-βH1 cells was adapted from a recent protocol^17^. A mixture of edited and un-edited cells is generated by this protocol, and currently there is no protocol to generate single clones in EndoC-βH1 cells. Specifically, 6 sgRNA sequences were designed from CRISPOR.org^49^ and cloned into plentiCRISPRv2 vector^50^ (Addgene catalog no. 52961). The sgRNAs are designed to target three CTCF motifs (2 sgRNA sequences for each motif) in OREG0020670, at the 5’-end, middle and 3’-end of the element. The non-targeting control gRNA sequence is from Addgene Plasmid no. 80262^51^. The lentiviral vectors were produced in Lenti-X 293T cells (Takara catalog no. 632180). For 80% confluency cells on one T175 flasks, a mixture of pMD2.G (6.85 µg) (Addgene catalog no. 12259), psPAX2 (10.3 µg) (Addgene catalog co. 12260), the respective cloned plentiCRISPRv2 (12.85 µg) were for transfection with 100 µL Lipofectamine LTX reagent (Invitrogen catalog no. 15338100). Growth medium was changed after 6 h. The supernatant containing viral particles was collected 48 h after transfection. Fresh medium was added in and the supernatant was collected again after 24 h. The viral particles were collected with Beckman Optima XL-100K ultracentrifuge at 29,000 rpm for 2 h at 4 °C. The virus pellet was resuspended in 1.5% BSA in phosphate buffered saline (PBS), and stored at −80 °C until use. EndoC-βH1 cells were transduced at a multiplicity of infection (MOI) of 8, and the stable CRISPR-edited EndoC-βH1 cells were generated after 4 ng/µl puromycin selection for 7 days. 8 ng/µL protamine was added into the medium during the lentiviral infection procedure. The CRISPR-editing efficiency was estimated by GeneArt™ Genomic Cleavage Detection Kit (Invitrogen catalog no. A24372).

### 3C library preparation

The 3C library was prepared similar to our previous publications^1,8^. Specifically, 5 × 10^7^ EndoC-βH1 cells were trypsinized and resuspended in 10 mL growth medium, then fixed by 2% final concentration formaldehyde for 10 min at room temperature. The reaction was stopped by adding 2.5 M glycine to a final concentration at 0.125 M for 5 min at room temperature. The cells were collected by centrifugation and resuspended in lysis buffer (10 mM Tris–HCl, pH 8.0, 10 mM NaCl, 0.2% NP-40), and ruptured by a Dounce homogenizer on ice. The cross-linked nuclei was collected by centrifugation, and digested by BglII (New England Biolabs, catalog no. R0144) with the following procedure: resuspending 5 × 10^7^ nuclei in 500 µL NEBuffer 3.1, and aliquoting 50 µL nuclei to 10 new tubes; for each new tube adding 312 µL NEBuffer 3.1, and 38 µL 1% sodium dodecyl sulfate (SDS) and incubating for 10 min at 65 °C; adding 44 µL 10% Triton X-100 and 400 U BglII; incubating overnight at 37 °C. The digested chromatin was re-ligated by T4 DNA ligase (New England Biolabs, catalog no. M0202L) with the following procedure for each aliquot: adding 86 µL 10% SDS and incubate for 30 min at 65 °C; transferring the liquid to a 50 mL conical tube; adding 745 µL 10% Triton X-100, 745 µL 10× ligation buffer (500 mM Tris–HCl, pH 7.5, 100 mM MgCl_2_, 100 mM dithiothreitol), 80 µL 10 mg/mL BSA, 80 µL 100 mM ATP, 5960 µL dH_2_O; adding 7.5 µL T4 DNA ligase and incubating overnight at 16 °C. The chromatin was de-crosslinked and digested with proteinase K (New England Biolabs, catalog no. P8107S) with the following procedure for each aliquot: adding 50 µl 10 mg/mL proteinase K in 1× TE buffer (10 mM Tris-HCl, pH 8.0, 1 mM EDTA), and incubating overnight at 65 °C; adding an additional 50 µL 10 mg/mL proteinase K in 1× TE buffer, and incubate for 2 h at 42 °C. The 3C library was purified by phenol/chloroform extraction (8 mL each) followed by ethanol precipitation (0.1 volume 3 M sodium acetate, pH 5.3 and 2.5 volume 100% ethanol, 2 h at −80 °C). The pellet was redissolved in 400 µL 1× TE buffer, pH 8.0, and was purified by phenol/chloroform extraction (400 µL each) followed by ethanol precipitation. The pellet was redissolved in 50 µL 1× TE buffer. After finishing preparation of all the aliquots, the samples were pooled together, and 1 µl 10 mg/mL RNase A (Thermo Scientific, catalog no. EN0531) was added following incubating the library for 15 min at 37 °C. The 3C library was stored at −80 °C before further experiments.

### Genotyping of 3C interactions

The genotypes of 3C interactions were determined by PCR amplification with PicoMaxx High Fidelity PCR Master Mix (Agilent, catalog no. 600650). The 3C primers used in this study were listed in Supplementary Table 3. In order to improve the specificity of the PCR reactions, 1% dimethyl sulfoxide (DMSO) was added when necessary. The 3C PCR product was either sequenced directly after purification with QIAquick Gel Extraction Kit (Qiagen, catalog no. 28704), or cloned into pGEM-T vector (Promega, catalog no. A3600) by TA-cloning. For TA-cloning, the PCR product was ligated with pGEM-T vector and transformed into *E. coli* DH5α cells. We usually randomly picked 12–24 colonies from the transformation agar plate and grew the *E. coli* culture in 10 mL LB Lenox medium overnight. The plasmids containing single cloned PCR product was extract with QIAprep Spin Miniprep Kit (Qiagen, catalog no. 27106), and was sent for Sanger sequencing to identify the alleles with BLAT^52^ alignment program (https://genome-asia.ucsc.edu/cgi-bin/hgBlat?command=start). The sequences without insert, with no signal, with non-specific PCR amplification product and dual signal (double colonies) were excluded from analysis. We reported the number of alleles identified in the procedure instead of their percentage.

### Chromatin immunoprecipitation (ChIP)

ChIP experiments on CTCF were carried out using the ChIP-IT Express Kit (Active Motif, catalog no. 53008). Briefly, 1.5 × 10^7^ cells were crosslinked by formaldehyde for 10 min at room temperature in the growth media, followed by 10 min incubation with glycine at room temperature to quench formaldehyde reactivity. Cells were washed twice with cold PBS, then the cell pellets were subjected to cell lysis by a Dounce homogenizer. The chromatin was sheared by a Bioruptor Plus sonication device to an average size of ~500 bp. The chromatin immunoprecipitation procedures were carried out according to the manufacturer’s instructions with an anti-CTCF antibody (Abcam, catalog no. ab70303), and 3 µL antibody was used in each experiment (1:33 dilution). The crosslink was reversed by heating, and the protein was removed by proteinase K digestion. PCR reactions were performed with PicoMaxx High Fidelity PCR Master Mix (Agilent, catalog no. 600650) using primers specified in Supplementary Table 3. The genotype of the PCR product was examined by direct sequencing or colony sequencing after cloning into pGEM-T vector. ChIP experiments of histone H3K9me3 and H3K27ac were carried out similarly. The anti-histone H3K9me3 antibody (Abcam, catalog no. ab8898, 1:50 dilution) and anti-histone H3K27ac antibody (Abcam, catalog no. ab4729, 1:50 dilution) were used.

### Methylation analysis

The genomic DNA of EndoC-βH1 cells was prepared by AllPrep DNA/RNA Mini Kit (Qiagen, catalog no. 80204). The bisulfite conversion was carried out with EpiTect Fast Bisulfide Kit (Qiagen, catalog no. 59802) for DMR in OREG0020670 and EZ DNA Methylation-Lightning Kit (Zymo Research, catalog no. D5030S) for *H19*-ICR HS5. The freshly converted genomic DNA was used as the template for the PCR reactions using EpiTaq HS DNA polymerase (Takara, catalog no. R110B). The primers are listed in Supplementary Table 3. The amplification for DMR was achieved under the conditions that favoring elongation (3 mM Mg^2+^, 0.2 mM dNTP, 1 µM primers and 2 min extension time). The PCR product was cloned into pGEM-T vector, and the individual clones were sequenced with a procedure similar to the 3C-PCR analysis above. The sequence analysis was performed with NCBI BLAST (https://blast.ncbi.nlm.nih.gov/Blast.cgi)^53^.

### Genotyping *INS-IGF2* isoform 1

Total RNA was extract from EndoC-βH1 cells by NucleoSpin RNA XS kit (Clontech, catalog no. 740902). The cDNA library was prepared from 5 µg total RNA by Maxima H Minus Reverse Transcriptase (Thermo Scientific, catalog no. EP0751) following the recommended condition for long RT-PCR (> 5 kb). The PCR product to genotype *INS-IGF2* isoform 1 was amplified by KAPA HiFi HotStart polymerase (KAPA Biosystems, catalog no. KM2605). Due to the high GC content of the template, 5% DMSO was added to the reaction. The product was verified after cloning into pGEM-T vector and sequenced by Sanger sequencing. The genotyping of the isoform was performed by direct sequencing the PCR product after agarose gel-purification.

### CTCF gel-shift chemiluminescent EMSA assay (electrophoretic mobility shift assay)

The CTCF gel-shift chemiluminescent EMSA assay was carried out with the Gel-shift Chemiluminescent EMSA Assay Kit (Active Motif, catalog no. 37341). The DNA oligo (purchased from Eurofins Genomics) sequence used was 5’-gtagtgacccCGgagccaCG*tggagccaccttcagaccca*gagctgaagcaggagggtgacCGg-3’. The biotin-dT label was on the 5’ end of the reverse strand. The CpGs that were either un-methylated or fully methylated are in underlined capitals. The CTCF-binding motif is in italic font. The EMSA assay was carried out following manufacture’s instruction with some modifications. The final concentration for the labeled dsDNA was 2 nM. NP-40 was added to the final concentration of 0.02%. 50 ng/µL Poly d(I-C) was used as a non-specific competitor. Maltose-binding protein-fused (MBP-fused) CTCF zinc finger 1–11 protein was over-expressed in *E. coli* and purified with amylose resin (New England Biolabs, catalog no. E8021S) according to the manufacturer’s protocol. The details of the CTCF preparation procedure were described in a previous publication^54^.

### Quantitative RT-PCR (qRT-PCR)

To assess the effect of OREG0020670 on *INS* expression in EndoC-βH1 cells, qRT-PCR experiments were performed on CRISPR-edited cells and control non-targeting gRNA treated cells, with Power SYBR™ Green Cells-to-CT™ Kit (Invitrogen catalog no. 4402954). *GAPDH* (glyceraldehyde-3-Phosphate dehydrogenase) endogenous control gene was used to normalize gene expression by the ΔΔCt method.

### Human pancreatic islet

The human pancreatic islets sample was provided by the Integrated Islet Distribution Program (IIDP, catalog no. Pancreas-U.Miami-4287_Islets, RRID:SAMN13866285) at City of Hope National Medical Center, which is funded by the National Institute of Diabetes and Digestive and Kidney Diseases (NIDDK), the National Institutes of Health (NIH Grant #2UC4DK098085). The islet donor was a 30-years old non-diabetic female (https://www.ncbi.nlm.nih.gov/biosample/SAMN13866285). The genomic DNA was extracted by Wizard Genomic DNA Purification Kit (Promega, catalog no. A1120). The *IGF2* locus was genotyped by PCR amplification with PicoMaxx High Fidelity PCR Master Mix (Agilent, catalog no. 600650). The total RNA was extracted by RNeasy Mini Kit (Qiagen, catalog no. 74014). The allele-biased expression of *IGF2* isoform 1 and *INS-IGF2* isoform 1 was determined by RT-PCR, following the procedures mentioned above for EndoC-βH1 cells. All islets shipped via the IIDP are derived from cadaver donors. The IIDP documents informed consent for research purposes from donor relatives prior to offering the de-identified islets to established laboratories engaged in islet research and registered with IIDP. Research at NIH on de-identified specimens is not considered human subjects research (NHSR). Thus, no special permissions are needed.

### Statistical analysis

Experimental data were analyzed using Graphpad QuickCalcs Web site (https://www.graphpad.com/quickcalcs/) and Microsoft Excel for Microsoft 365. Statistical comparison was conducted using the unpaired two-tailed Student’s *t* test. *P* < 0.05 was considered statistically significant. Data were shown as mean ± SD.

### Reporting summary

Further information on experimental design is available in the Nature Research Reporting Summary linked to this paper.

## Supplementary information

Supplementary Information

Reporting summary

## Data Availability

All data associated with this study are available from the corresponding authors upon reasonable request. Source data are provided with this paper. The 4C-seq data used in this study^1^ are available in the National Center for Biotechnology Information Gene Expression Omnibus (GEO) database under accession code GSE112346. Source data are provided with this paper.

## Source data

source data
