# Supplementary material for: Large parental differences in chromatin organization in pancreatic beta cell line explaining diabetes susceptibility effects
Source: Nat Commun. 2021 Jul 15;12:4338. doi: 10.1038/s41467-021-24635-2 (PMC8282625; doi:10.1038/s41467-021-24635-2)
Supplement: Supplementary file 1 — Supplementary Information [file 41467_2021_24635_MOESM1_ESM.pdf]

SUPPLEMENTARY INFORMATION

Large Parental Differences in Chromatin Organization in Pancreatic Beta Cell Line Explaining Diabetes Susceptibility Effects

Xing Jian[1] and Gary Felsenfeld[1]*

[1]Laboratory of Molecular Biology, National Institute of Diabetes and Digestive and Kidney Diseases, National Institutes of Health, Bethesda, MD 20892 USA

Supplementary Figure 1

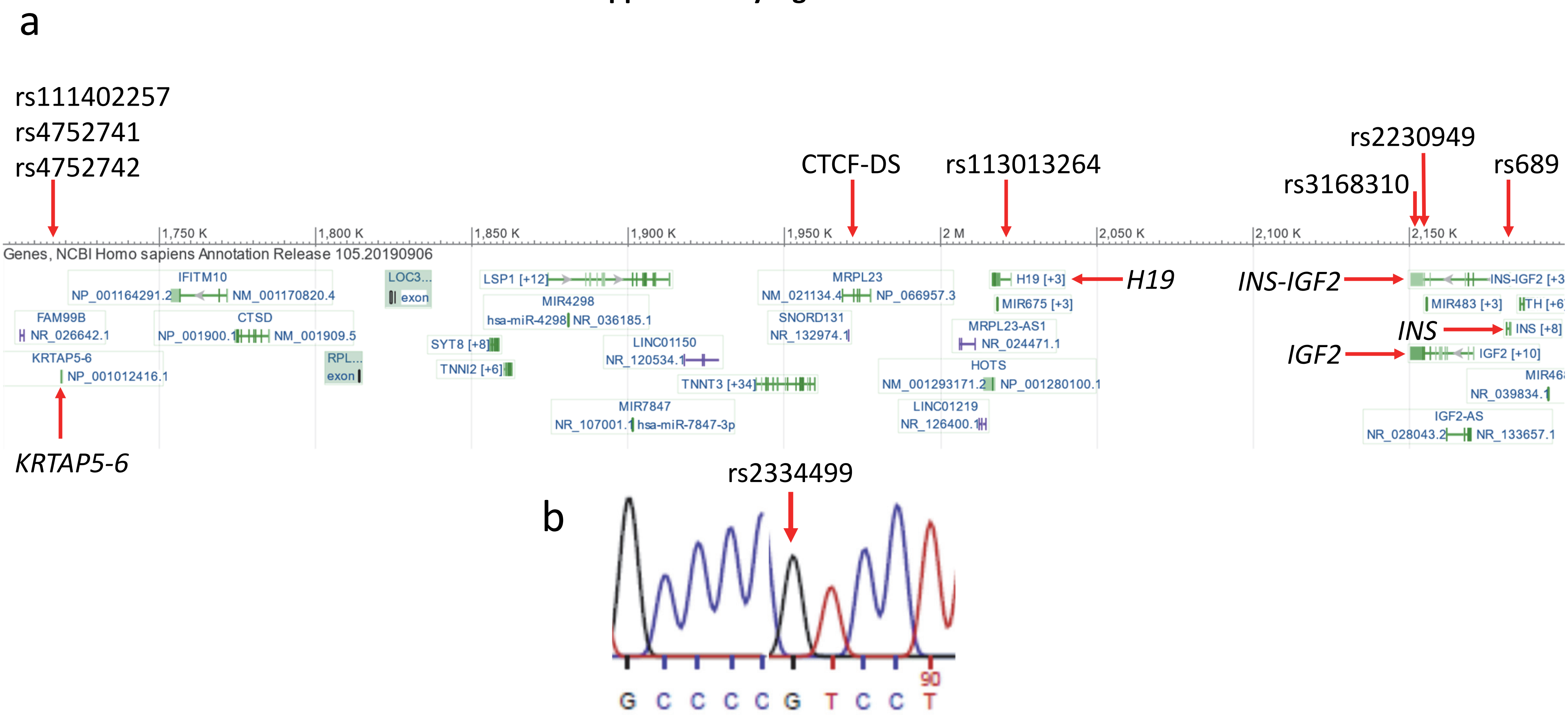

Supplementary Fig. 1. A detailed view of the human chromosome 11 (hg19 assembly) from 1.7 Mb to 2.2 Mb.

a The genetic features mentioned in this study are marked.

b Genotyping of rs2230949 from genomic DNA of EndoC-βH1 cells, showing C/C genotype. The sequencing primer reads from the reverse strand.

Supplementary Figure 2

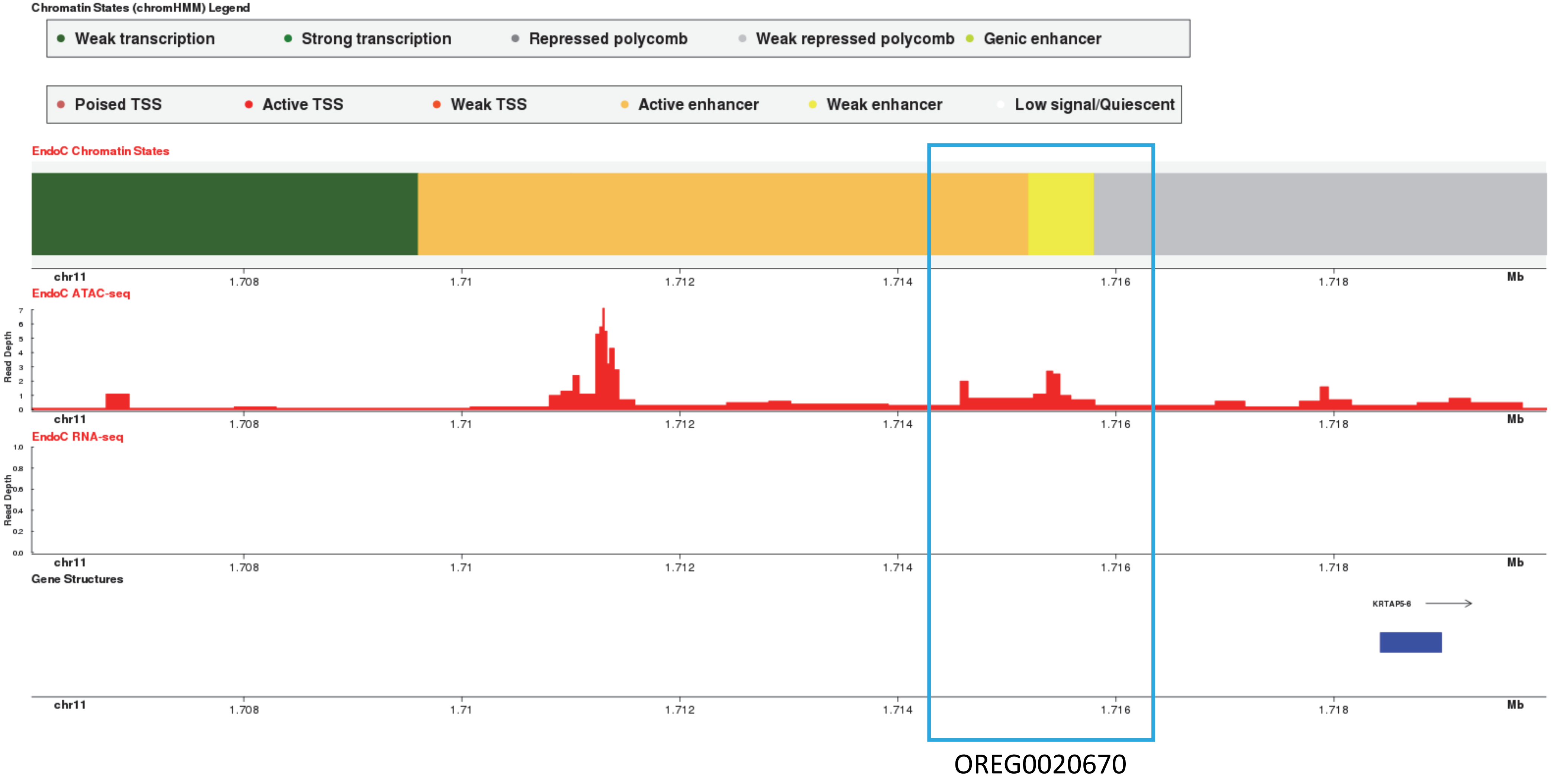

Supplementary Fig. 2. The chromatin state of element OREG0020670. Data is retrieved from R Shiny application for browsing human islet and EndoC-βH1 genomic data (https://shinyapps.jax.org/endoc-islet-multi-omics/)[1]. OREG0020670 is marked with a blue frame.

Supplementary Figure 3

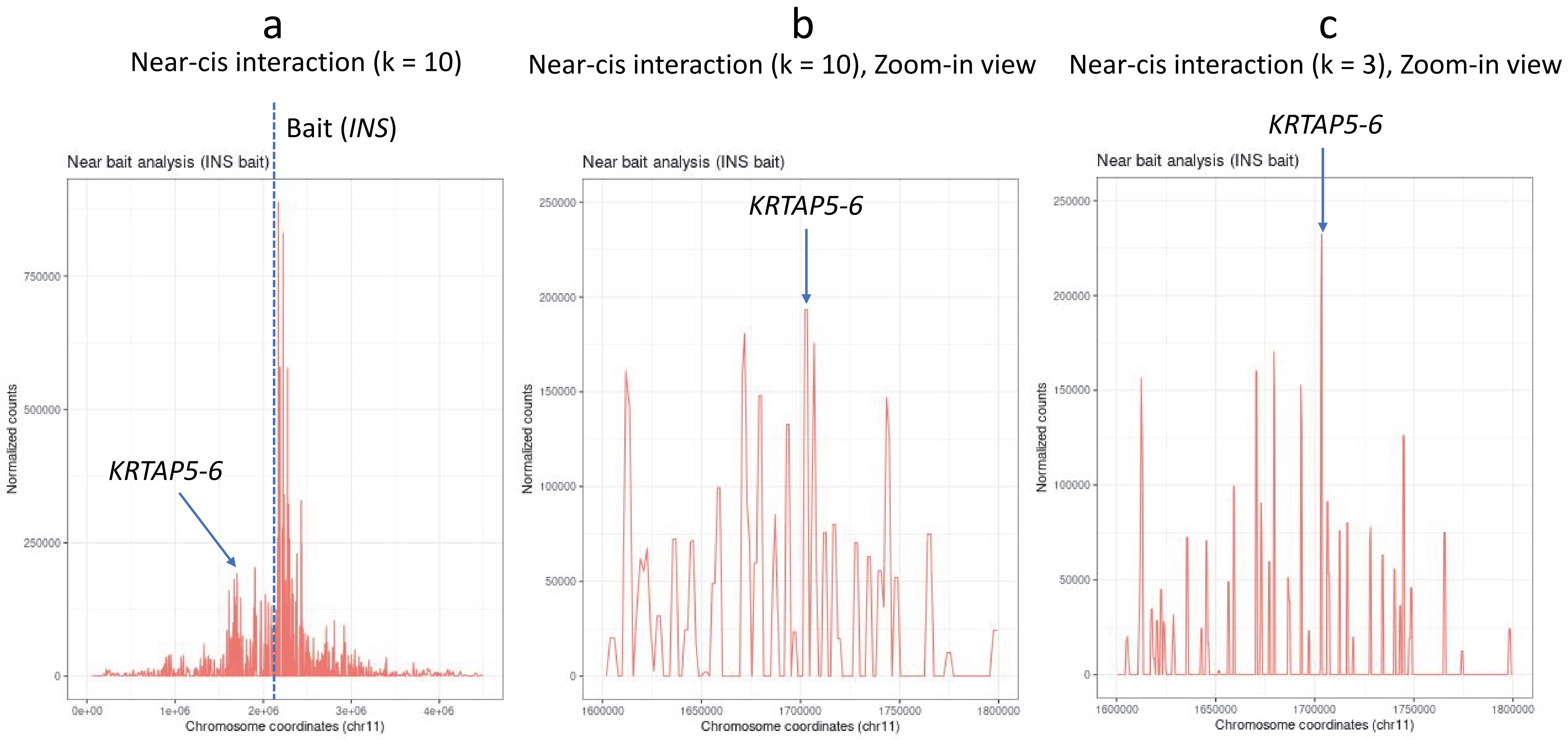

Supplementary Fig. 3. Normalized 4C-seq read counts from EndoC-βH1 using the *INS* promoter (blue dotted line) as the bait region[2].

a Contact map at chromosome 11, 1 – 4,500,000 bp with k = 10.

b Zoom-in view at chromosome 11, 1,600,000 – 1,800,000 bp with k = 10.

c Zoom-in view at chromosome 11, 1,600,000 – 1,800,000 bp with k = 3.

Supplementary Figure 4

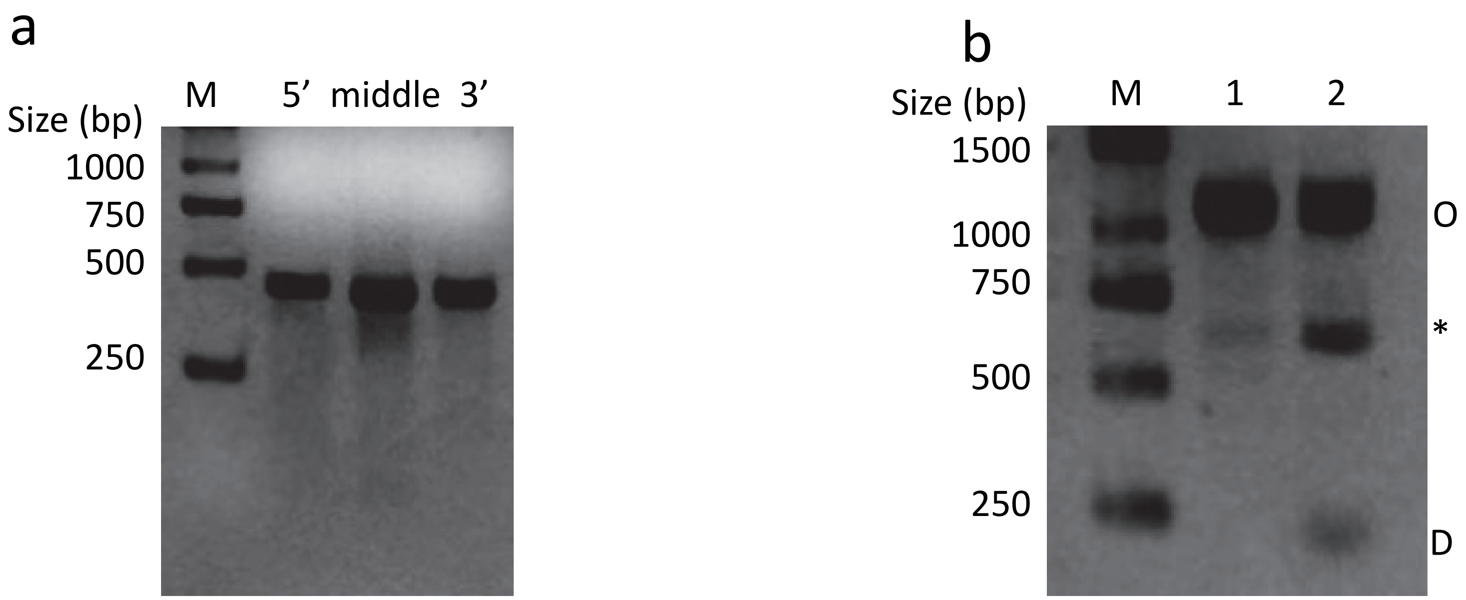

Supplementary Fig. 4. **Quantification of CRISPR/Cas9 editing efficiency by T7 endonuclease I-based genomic cleavage assay (a) and deletion sensitive PCR (b).** In the genomic cleavage assay, the on-target genome editing efficiency was measured by the cleavage of mis-matched PCR product from insertions and deletions generated by CRISPR/Cas9 editing. 5'-, middle- and 3'- CTCF sites are labeled above figure a. In figure b, lane 1 is product from unedited genomic DNA, lane 2 is the product from edited genomic DNA. M: DNA marker. O: original product. D: product with deletion. *: non-specific amplification product. Source data are provided as the Source Data file. The figures are representative of 3 biological independent repeats.

Supplementary Figure 5

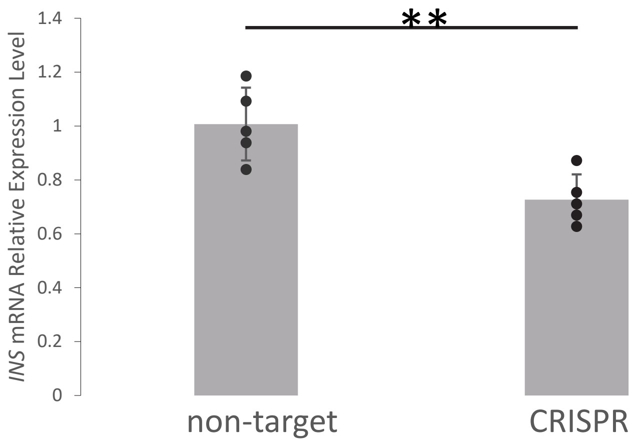

Supplementary Fig. 5. The effect of OREG0020670 on *INS* mRNA expression, assayed by qRT-PCR. *n* = 5 biologically independent samples, unpaired two-sided t-test, *p* = 0.0042 (indicated with “**” for $p < 0.01$). Data are presented as mean values +/- SD. *GAPDH* gene was used as the internal control. Source data are provided in the Source Data file.

Supplementary Figure 6

hg19. chr11:1715431 - 1716230

ggcagctctgacccctcccgctgcgctcctccagccgcatccctgcagcc
ttgggccctactggcccggggggaggaggcactgcgctttggggaggaaga
gagactaaaggggagaccgagaaccccgggtcaggaagcggagagctaaa
acgacaaccgtgggtcagcggaggccggcaacttcatttcatgacctctg
gcctccaaagcagctctgcaaagtggtcaatgctcctctcgttgtgtaga
tgaagatactgagggccagggaggctgtggactgcctgatgtcacacgac
tggtggggcggcgggcgggtctgtccccaggcgattgtcctcgaggcaca
cggcggggcagagcttggaagcggccgaggtgggggtccctgggtgtgga
tgtagtgacccggagccacgtggagccaccttcagacccagagctgaag
caggagggtgaccggtagagcaaggccgtgcacaccccgttgcagaatt
gaaaacgcgcctcaaaataaaaccccgtgtttcacaagaatgcactaatt
cccgaagatatccgggcgcacgggatgcagtggggattggagatgcaggg
gccatgcaagtgagaaggaccctgctcagcgtgaggctctgatggtcttg
aattcctttgccatgctcggacaaggggcctgcgctgtcacgttgctttg
gaccctgaaaattcgggagcccgtcctgagcccacgctgccatagttgcc
aggtatccagcatatgcacaaaacacctccacgccggtgctctgccctgc

Unmethylated CpG

Partially methylated CpG mentioned in Reference 3.

CpG in DMR

CTCF binding motif

Methylated CpG

3 SNPs for genotyping
C,G,A / -C A G

Supplementary Fig. 6. Detailed sequences showing methylation pattern of OREG0020670 in EndoC-βH1 cells. Color of the CpGs indicates the methylation status. Purple: unmethylated. Green: methylated. Orange: partially methylated without parental-of-origin bias. Red: differentially methylated with parental-of-origin bias. Cyan: SNPs used for genotyping. Pink: CTCF binding motif. Underlined: primers for ChIP-PCR.

Supplementary Figure 7

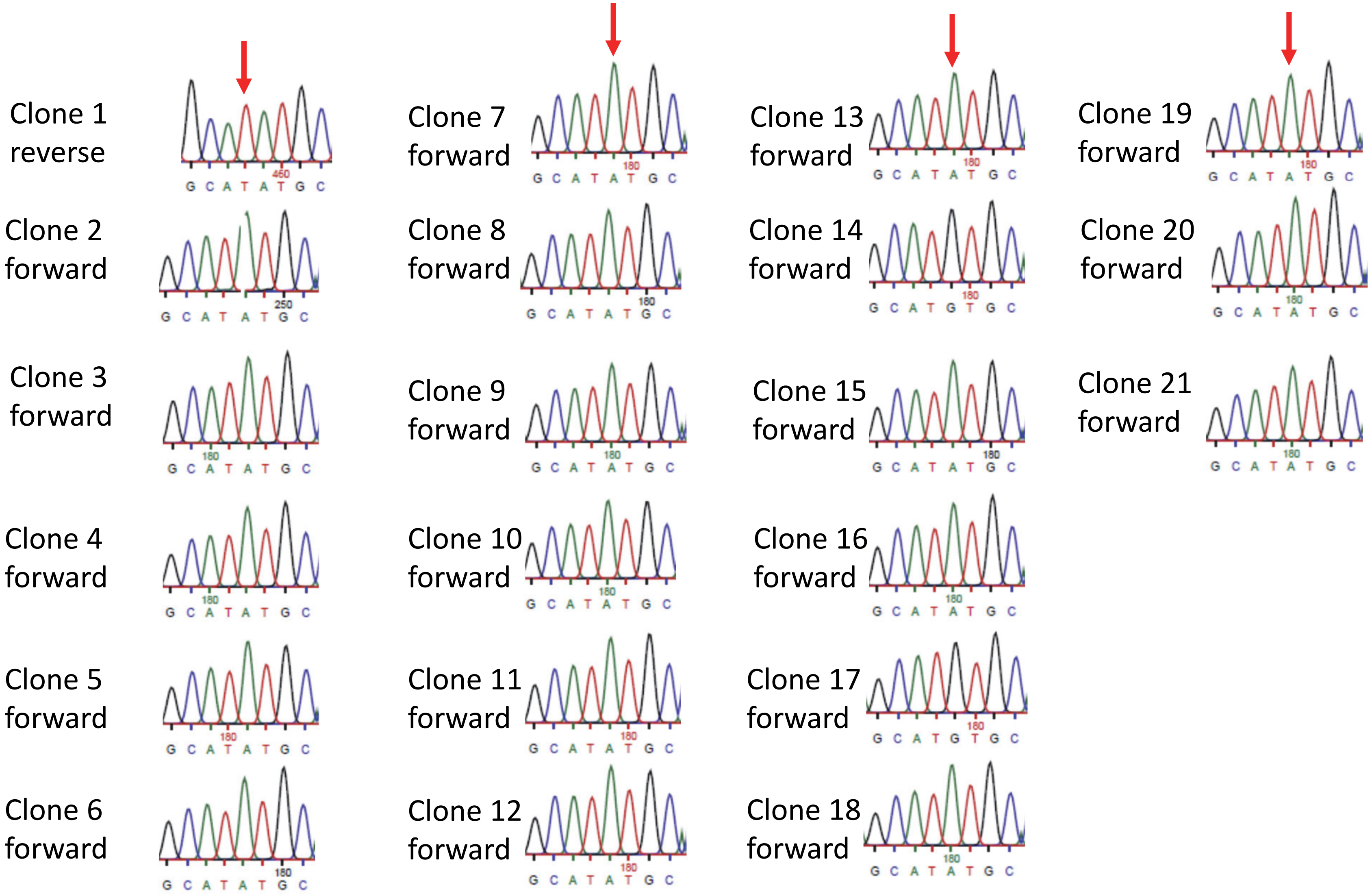

Supplementary Fig. 7. Sanger sequencing results showing the allele of rs4752742 in 21 clones of *INS*-OREG0020670-3C-PCR product in T-vector. Except for clone 1, the sequencing read is from the forward strand. The red arrows indicate the location of rs4752742.

Supplementary Figure 8

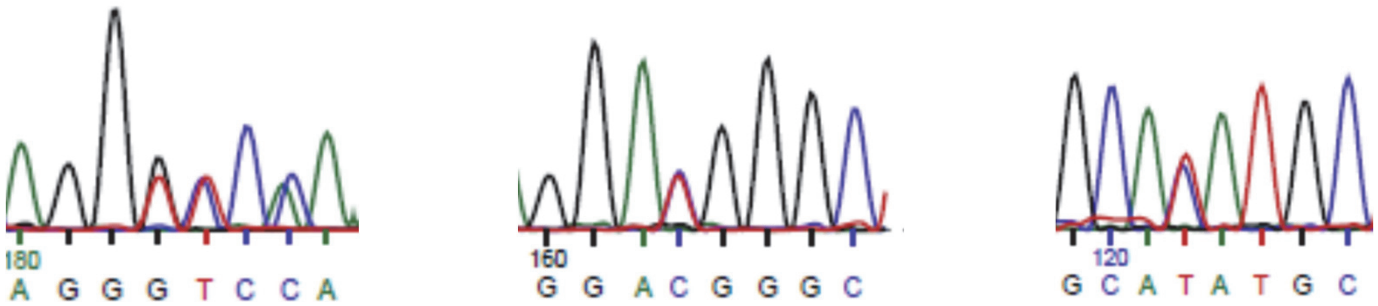

rs111402257: C, 52%; -C, 48% **rs4752741: G, 50%; A, 50%** **rs4752742: A, 54%; G, 46%**

Supplementary Fig. 8. Control PCR for 3C analysis in Fig. 2a from a mixture of 1:1 heterozygotic template.

Supplementary Figure 9

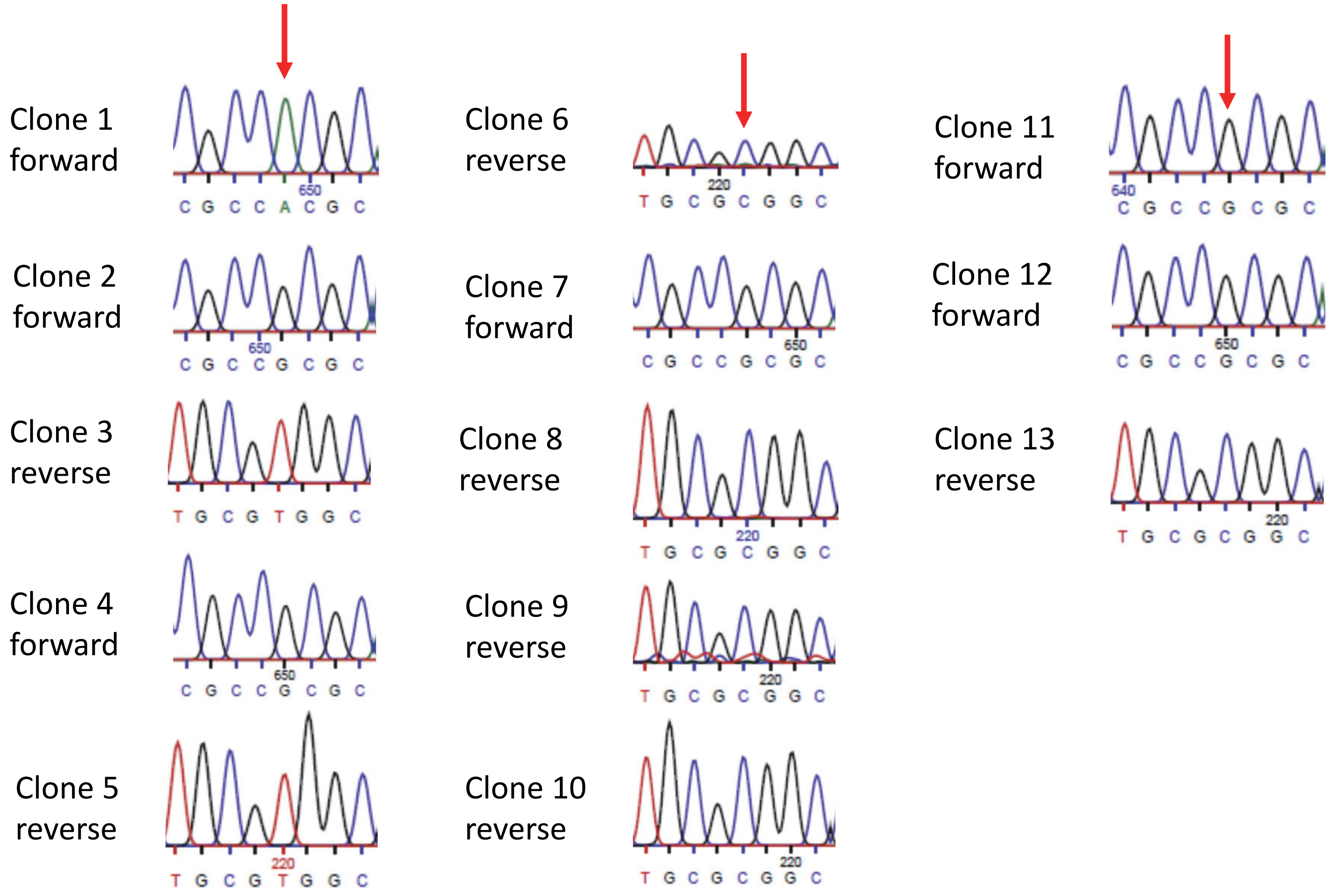

Supplementary Fig. 9. Sanger sequencing results showing the allele of rs113013264 in 13 clones of *H19*-ICR-CTCF-DS-3C-PCR product in T-vector. The direction of sequencing read is specified for each clone. The red arrows indicate the location of rs113013264.

Supplementary Figure 10

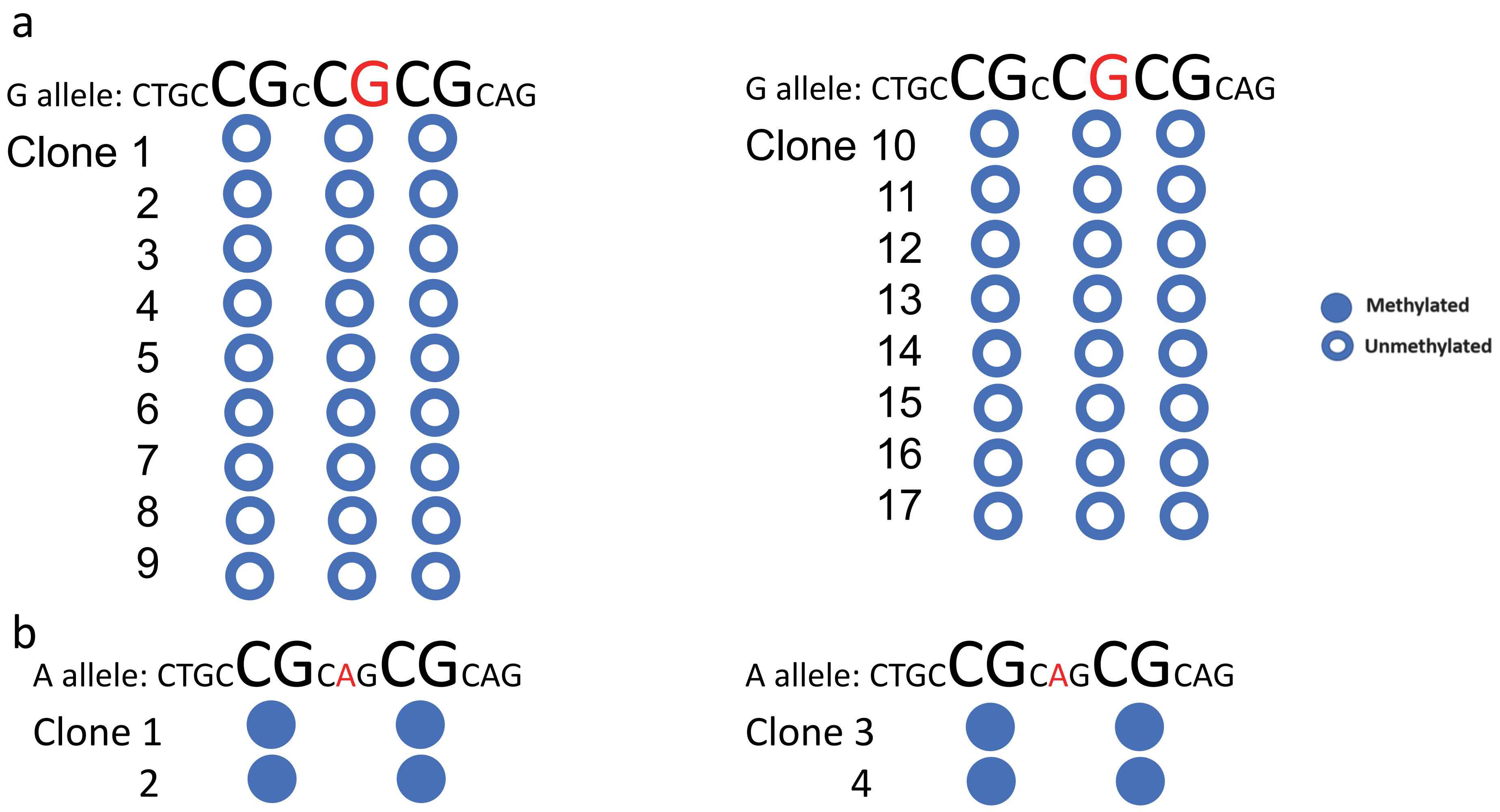

Supplementary Fig. 10. Bisulfite sequencing analysis of CTCF-binding site HS5 in *H19*-ICR locus. CpGs are highlighted with larger font size. SNP rs113013264 is highlighted in red. The G allele is shown in (**a**), and the A allele is shown in (**b**).

Supplementary Figure 11

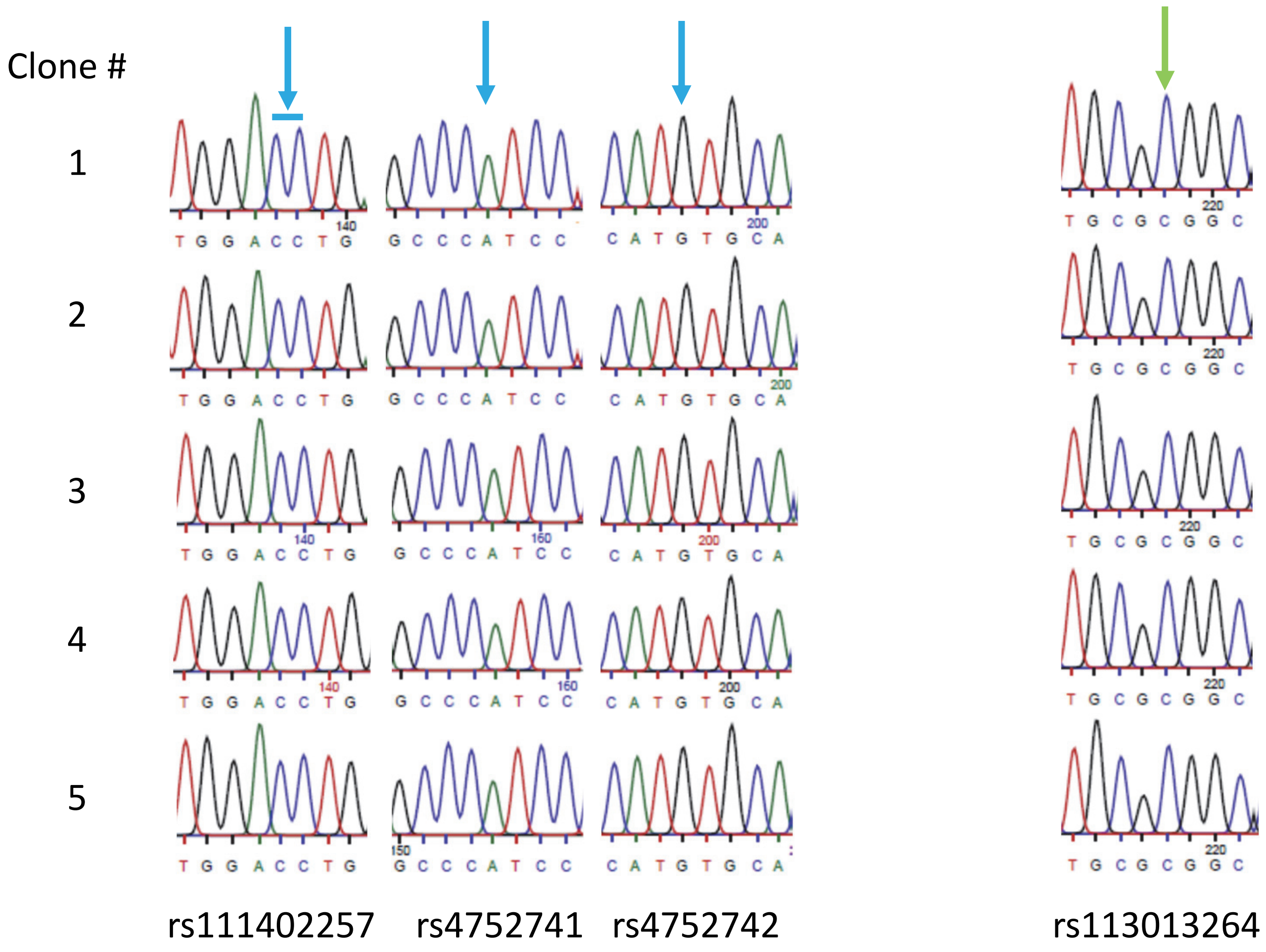

Supplementary Figure 11 (cont.)

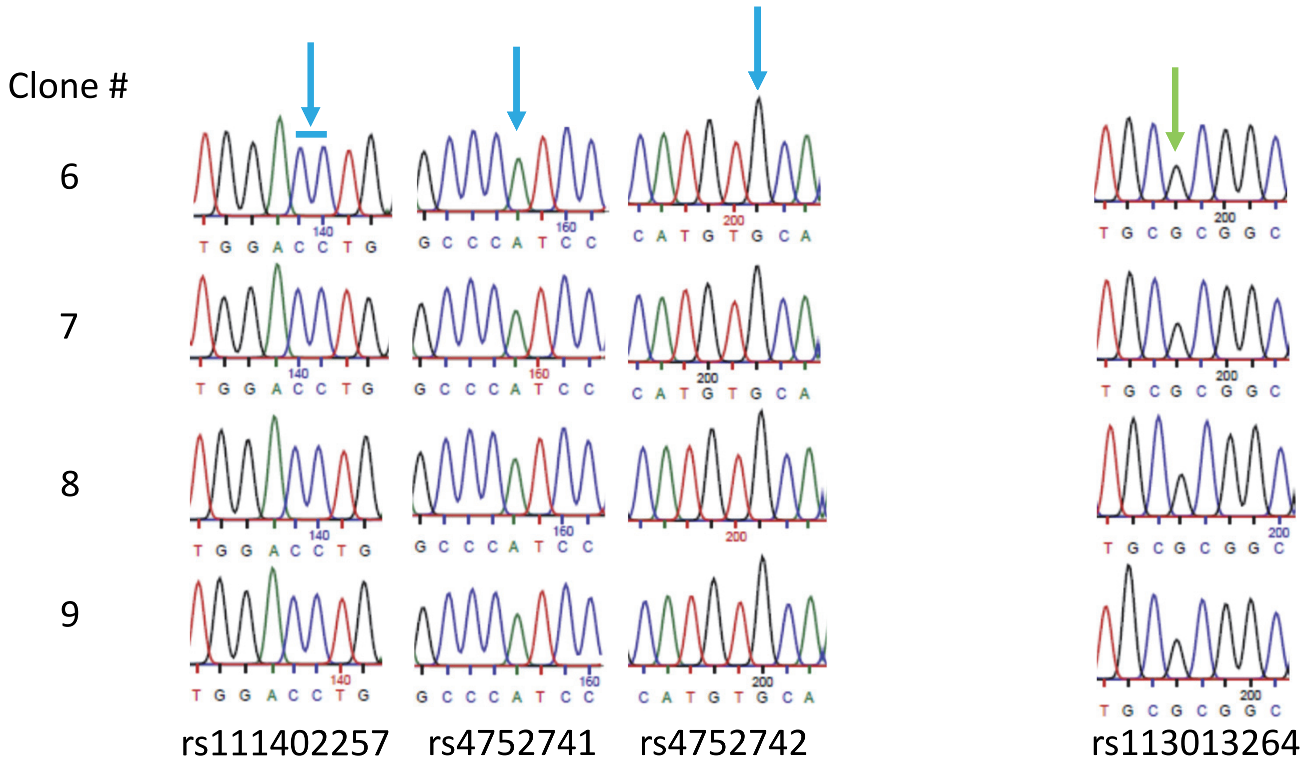

Supplementary Fig. 11. Sequencing results of eight additional single clones of 3C-PCR product between *H19*-ICR and OREG0020670 near the *KRTAP5-6* gene showed that it contains G allele (marked with green arrow) of *H19*-ICR (rs113013264) and -C/A/G allele (marked with blue arrows) of OREG0020670 (rs111402257/ rs4752741/rs4752742). The sequencing read is from the reverse strand for *H19*-ICR and from the forward strand for OREG0020670.

Supplementary Figure 12

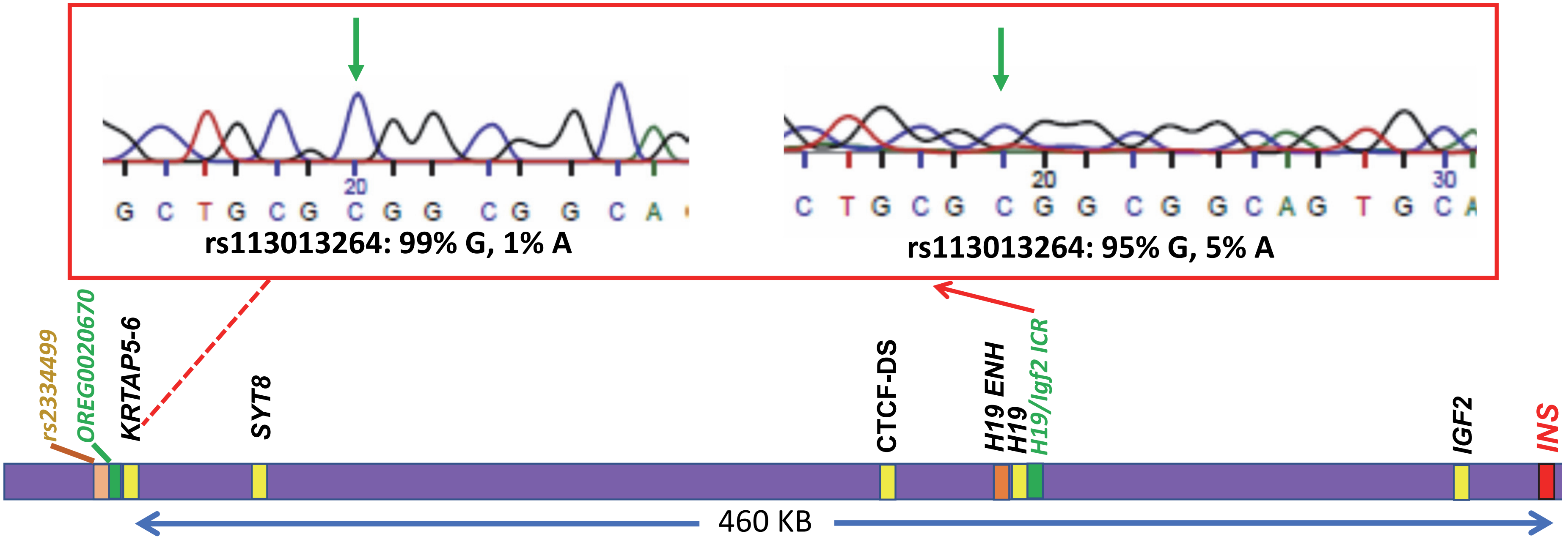

Supplementary Fig. 12. The contact between *H19*-ICR and OREG0020670 near the *KRTAP5-6* gene is enriched with the G allele of rs113013264 over the A allele in two different 3C ligation products. The sequencing read is from the reverse strand. Red arrow and dashed line show contact.

Supplementary Figure 13

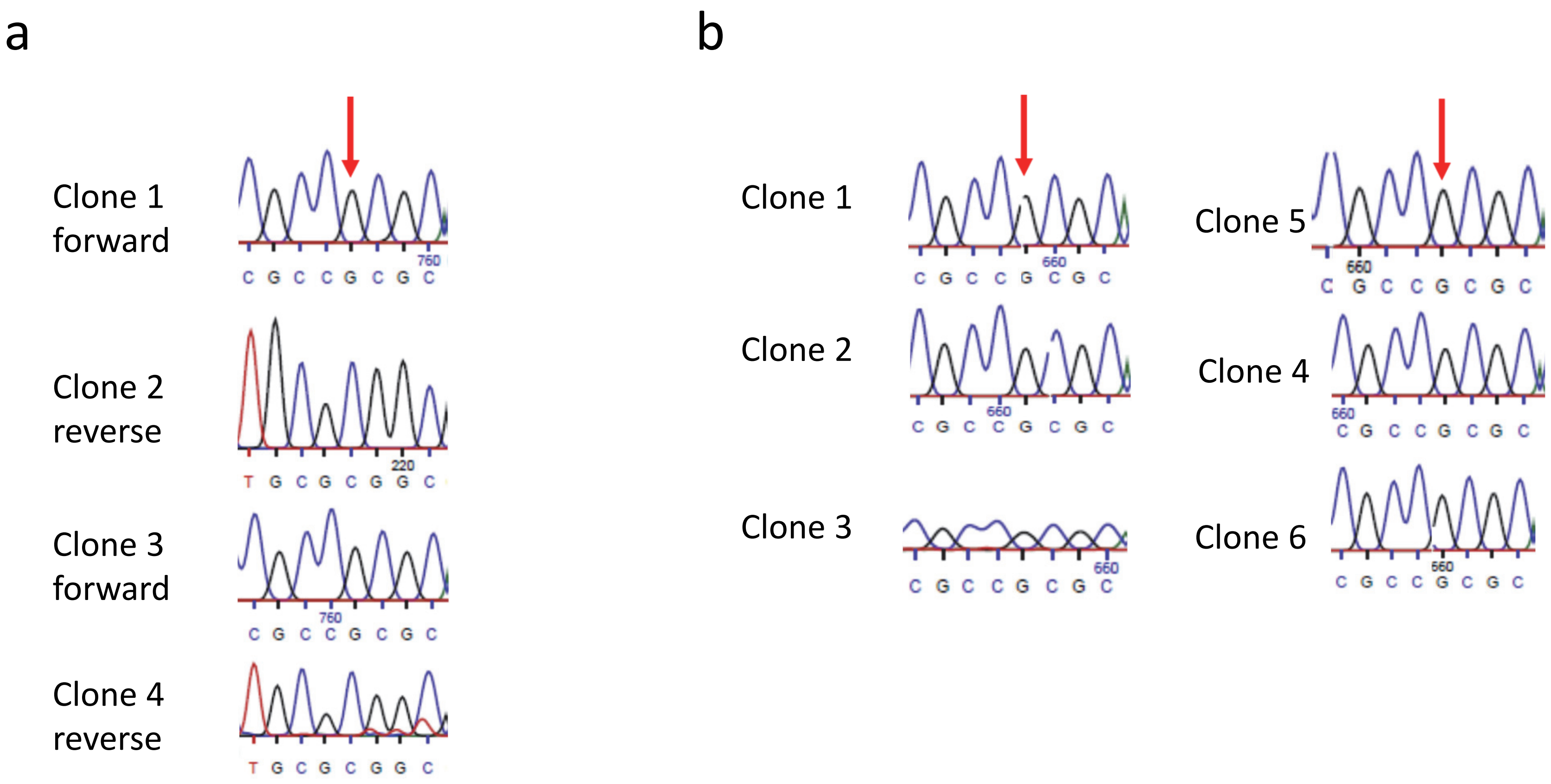

Supplementary Fig. 13. Sanger sequencing results showing the allele of rs113013264 in 10 clones of *H19*-ICR-OREG0020670-3C-PCR product in T-vector. The red arrows indicate the location of rs113013264.

a PCR product with primer set 1. The direction of sequencing read is specified for each clone.

b PCR product with primer set 2. The sequencing read is from the forward strand.

Supplementary Figure 14

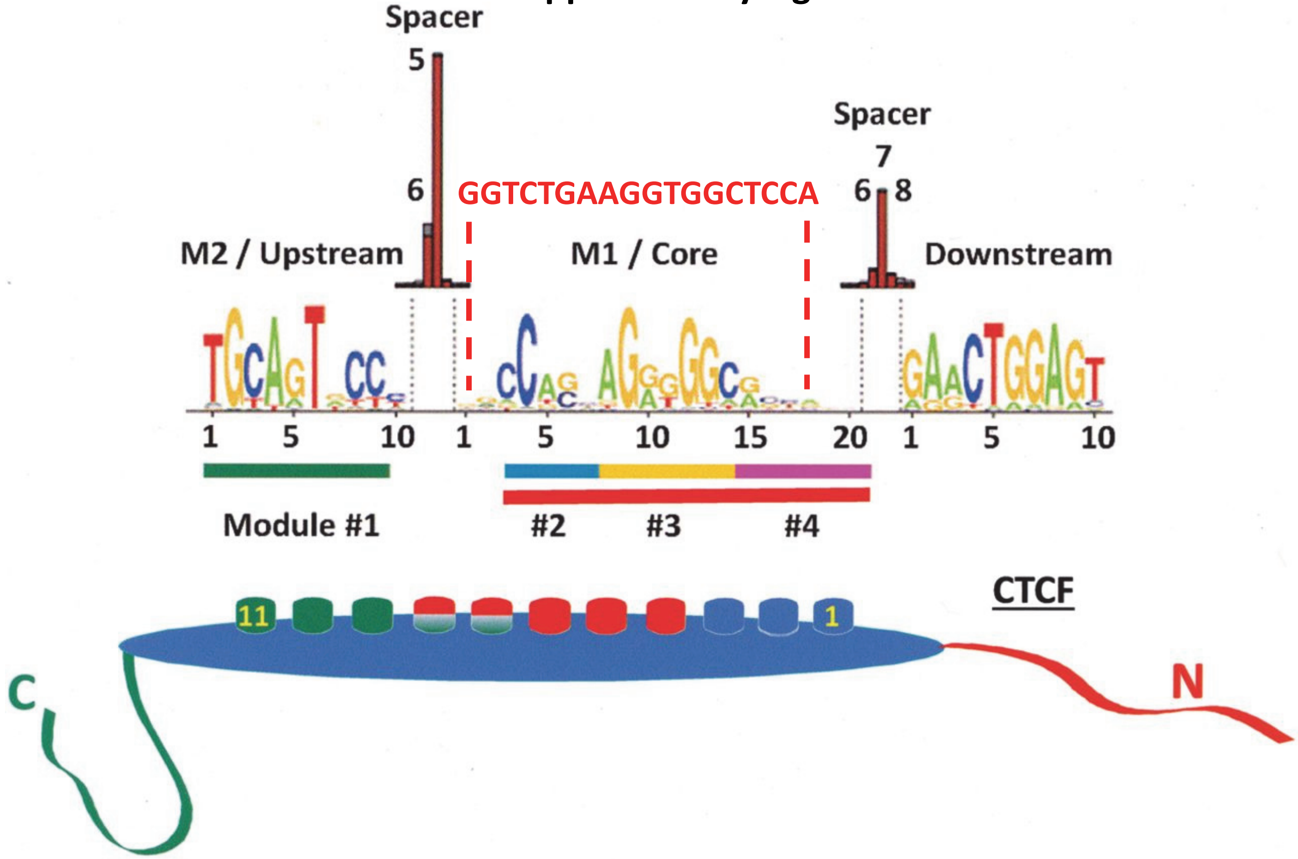

Supplementary Fig. 14. Identified CTCF motif (highlighted in red) in DMR of OREG0020670, compared with the canonical CTCF motif. The figure is modified from a previous publication of our group[4].

Supplementary Figure 15

a

Fully Methylated DNA sequence:

5’-gtagtgaccc**(5-me-c)g**gagcca**(5-me-c)g***tggagccaccttcagaccca*gagctgaagcaggagggtgac**(5-me-c)g**g-3’

Unmethylated DNA sequence:

5’-gtagtgaccc**cg**gagcca**cg***tggagccaccttcagaccca*gagctgaagcaggagggtgac**cg**g-3’

b

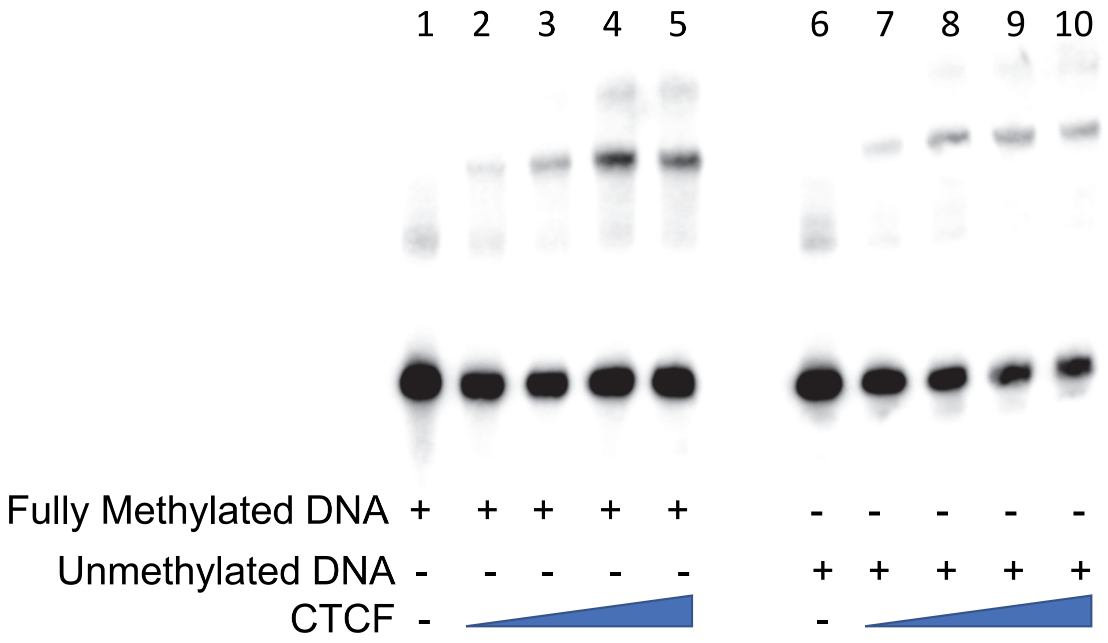

Supplementary Fig. 15. Gel-shift chemiluminescent EMSA assay testing the effect of methylation on CTCF binding to DMR sequence shown in Supplementary Fig. 6.

a The sequences of the dsDNA used in the assay. The CpGs are underlined, and the CTCF motif is highlighted in red.

b CTCF zinc finger 1-11 fragment binds to both fully methylated and unmethylated DMR dsDNA. Lane 1-5: methylated DNA. Lane 6-10: unmethylated DNA. Lane 1 and 6: no CTCF added. Lane 2 to 5 and 7 to 10: increasing amount of CTCF added. Biotinylated dsDNA is visualized on the blot. The figure is representative of 2 biological independent repeats.

Supplementary Figure 16

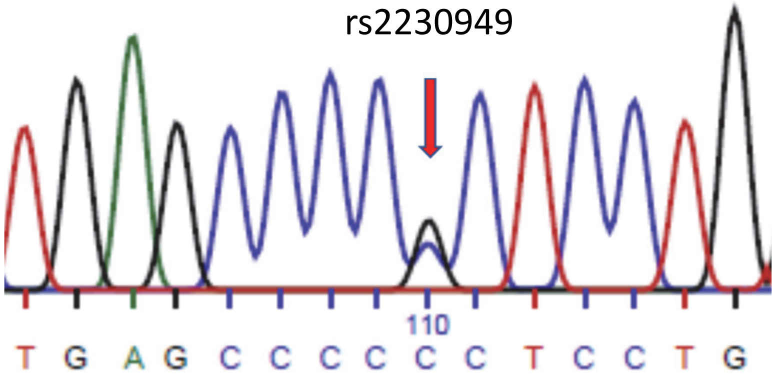

Supplementary Fig. 16. Genotyping of rs2230949 from genomic DNA of EndoC-βH1 cells, showing G/C genotype. The sequencing primer reads from the reverse strand.

Supplementary Figure 17

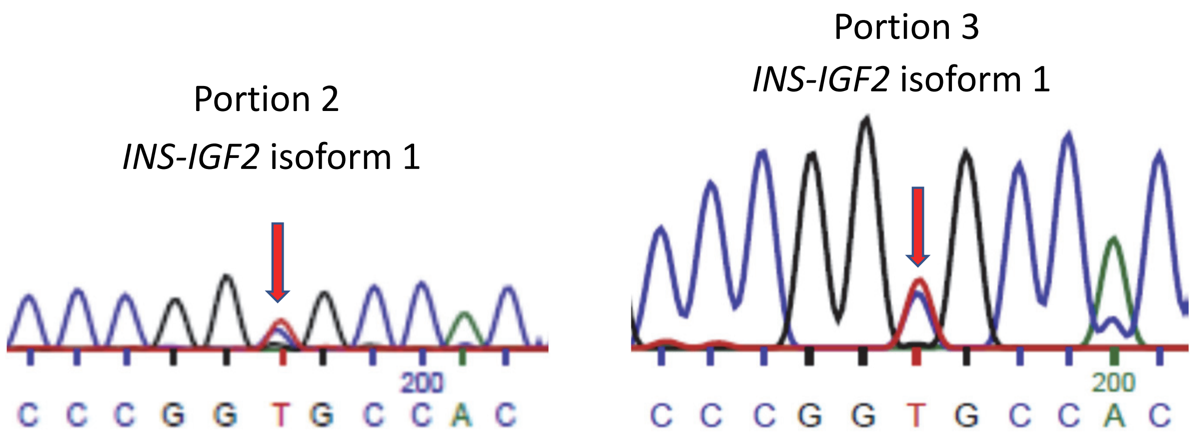

Supplementary Fig. 17. Genotyping of rs2230949 of *INS-IGF2* isoform 1 transcript in two additional portions of the human islet sample. The expression is biased toward the A allele in these two portions as well. The sequencing primer reads from the reverse strand.

Supplementary Figure 18

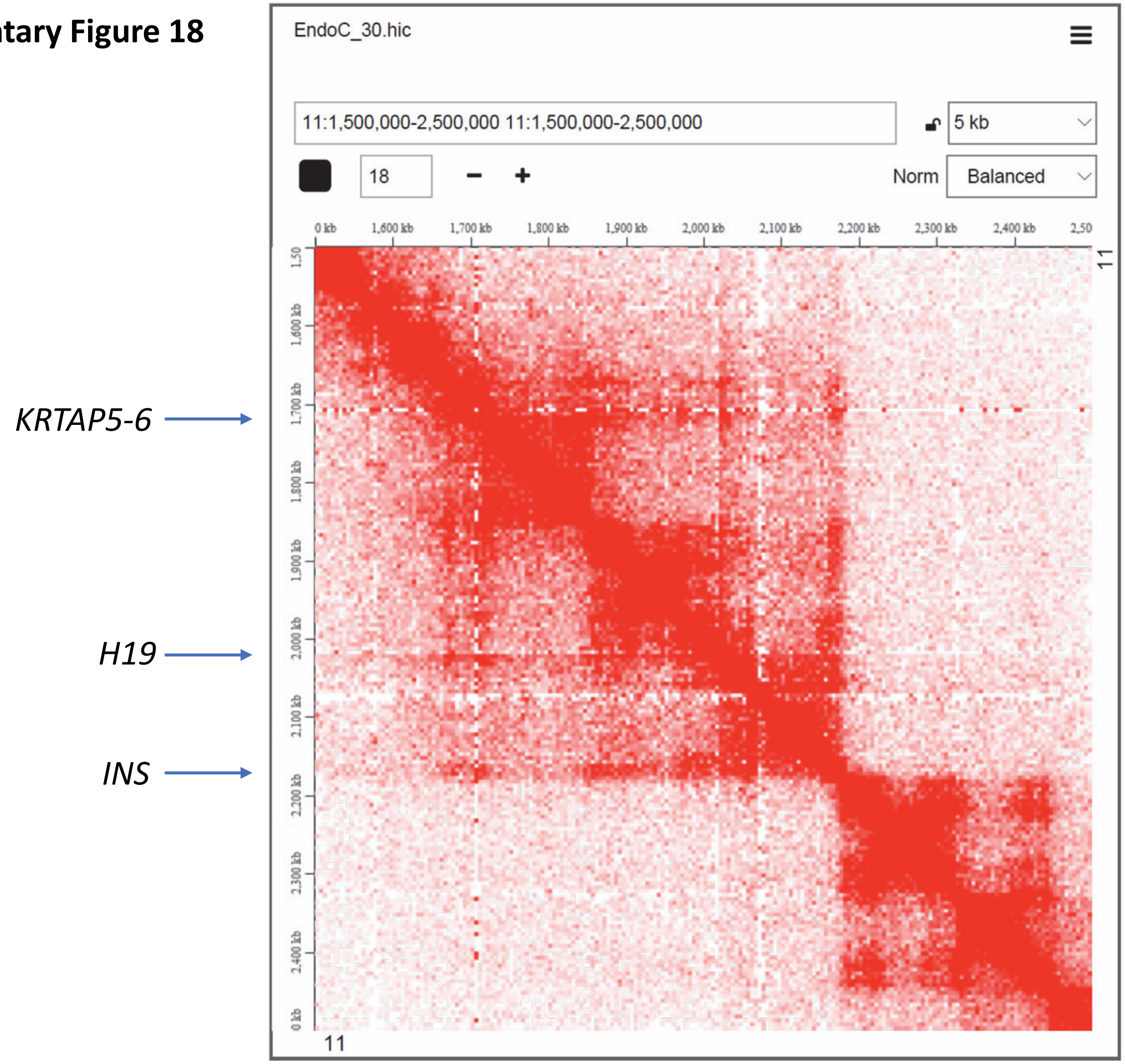

Supplementary Fig. 18. Juicebox Hi-C map of EndoC-βH1 cells from hg19 chromosome 11 1.5 Mb to 2.5 Mb at 5 kb resolution[1], showing *INS* gene at a domain boundary.

Supplementary Figure 19

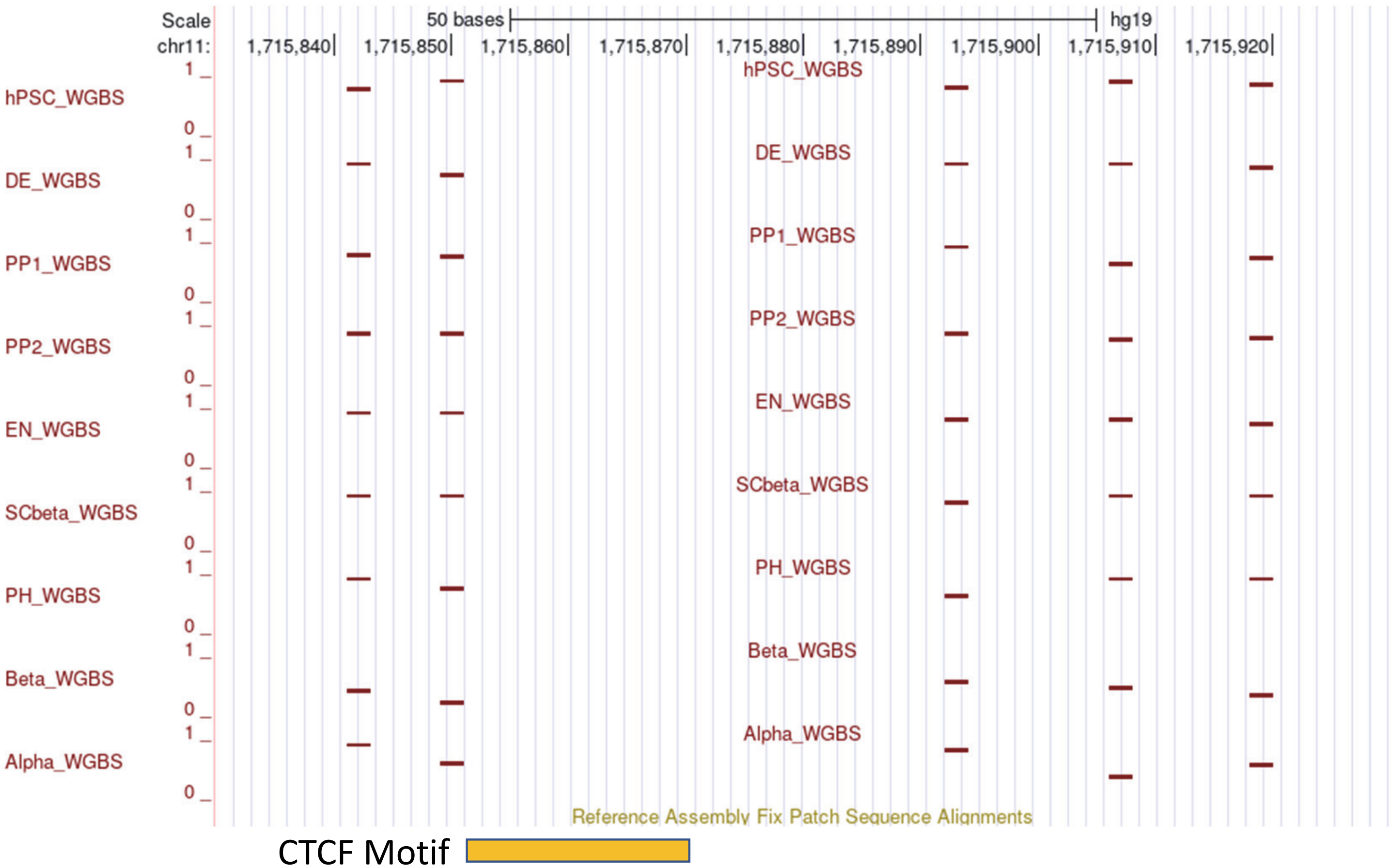

Supplementary Fig. 19. UCSC Genome Browser screenshot (http://genome.ucsc.edu) showing methylation status of the identified DMR (red CpGs in Supplementary Fig. 6) in human cells related to islet maturation process. The dataset is from http://meltonlab.rc.fas.harvard.edu/data/pancreatic_enhancers/[5]. Y-axis: DNA methylation level (0-1). hPSC: human pluripotent stem cells. DE: definitive endoderm cells. PP1: $PDX1^+$ pancreatic progenitor cells. PP2: $PDX1+NKX6.1^+$ pancreatic progenitor cells. EN: endocrine progenitor cells. SCbeta: stem-cell derived β-cells. PH: $insulin^+$ $glucagon^+$ polyhormonal cells. Beta: primary β-cells. Alpha: primary α-cells. Yellow bar at the bottom: CTCF motif.

Supplementary Figure 20

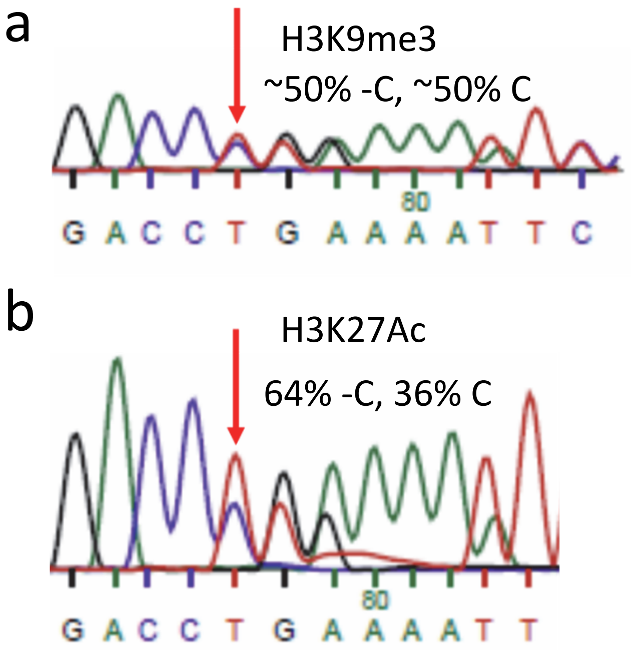

Supplementary Fig. 20. SNP typing of H3K9me3 and H3K27ac at the DMR region. The red arrows indicate the location of rs111402257. The sequencing primer reads from the forward strand.

a Sanger sequencing result of PCR product of at DMR region including SNP rs111402257 from a H3K9me3-ChIP sample.

b Sanger sequencing result of PCR product of at DMR region including SNP rs111402257 from a H3K27ac-ChIP sample.

Supplementary Table 1. Parental-origin-specific analyses of diabetes-susceptibility at SNP rs2334499[3].

Disease, SNP [alleles]	Standard case-control test		Test of Association with parental origins					
T2D, rs2334499 [T/C]	OR	*P*	Paternal allele		Materal allele		Paternal vs maternal (case only)	
			OR	*P*	OR	*P*	n12:n21	*P*
1468 (discovery)	1.11	0.0017	1.41	4.3×10^{-9}	0.87	0.02	437:276	7.0×10^{-9}
783 (replication)	1.02	0.71	1.23	0.0055	0.84	0.023	222:157	8.0×10^{-4}
2251 (combined)	1.08	0.034	1.35	4.7×10^{-10}	0.86	0.002	659:433	4.1×10^{-11}

For details of the statistical analysis of the data, please refer to reference 3.

Supplementary Table 2. List of potential transcription factors that would bind DMR in OREG0020670.

latrix Fami	Detailed Family Information	Matrix	Detailed Matrix Information	Opt.	tart positic	nd positio	chor positi	Strand	ore sin	atrix sir	. sim. - c	Sequence
V$DEAF	Homolog to deformed epidermal autoregulatory factor-1 from D	V$NUDR.01	NUDR (nuclear DEAF-1 related transcriptional regulator protein)	0.75	13	31	22	-	1	0.82	0.07	accTCGGccgcttccaagc
V$ZF02	C2H2 zinc finger transcription factors 2	V$ZKSCAN3.01	Zinc finger with KRAB and SCAN domains 3	1	18	40	29	-	1	1	0	gggacCCCCacctcggccgcttc
V$GLIF	GLI zinc finger family	V$GLIS2.01	GLIS family zinc finger 2	0.86	24	40	32	-	1	0.918	0.058	gggaCCCCcacctcggc
V$EGRF	EGR/nerve growth factor induced protein C & related factors	V$WT1.01	Wilms Tumor Suppressor	0.92	26	44	35	+	1	0.928	0.008	cgaggTGGGggtccctggg
V$NFKB	Nuclear factor kappa B/c-rel	V$NFKAPPAB50	NF-kappaB (p50)	0.83	29	43	36	-	1	0.84	0.01	ccaGGGAcccccacc
V$PLAG	Pleomorphic adenoma gene	V$PLAGL1.02	Pleiomorphic adenoma gene-like 1 (secondary DNA binding preference)	0.81	29	51	40	+	1	0.867	0.057	ggtgGGGGtccctgggtgtggat
V$ZTRE	Zinc transcriptional regulatory element	V$ZTRE.01	ZTRE motifs (1 bp spacer), ZNF658 binding site	0.75	32	48	40	-	1	0.777	0.027	cacacccaGGGAccccc
V$ZTRE	Zinc transcriptional regulatory element	V$ZTRE.01	ZTRE motifs (1 bp spacer), ZNF658 binding site	0.75	34	50	42	+	0.848	0.757	0.007	gggtccctGGGTgtgga
V$KLFS	Krueppel like transcription factors	V$EKLF.02	Erythroid krueppel like factor (EKLF, KLF1)	0.93	37	55	46	+	1	0.975	0.045	tccctgGGTGtggatgtag
V$RXRF	RXR heterodimer binding sites	V$RXRA.01	Retinoid X receptor alpha homodimer, DR1 sites	0.83	48	72	60	-	1	0.911	0.081	acgtggctccggGGTCactacatcc
V$XBBF	X-box binding factors	V$MIF1.01	MIBP1 / RFX1 complex	0.76	52	70	61	-	0.8	0.776	0.016	gtggctccgggGTCActac
V$ESRR	Estrogen-related receptors	V$ESRRA.02	Estrogen-related receptor alpha (secondary DNA binding preference)	0.9	52	74	63	-	1	0.927	0.027	ccacgtggctccggGGTCactac
V$SMAD	Vertebrate SMAD family of transcription factors	V$GC_SBE.01	GC-rich Smad1/5 binding element	0.94	61	71	66	-	1	0.946	0.006	cgtggCTCCgg
V$EBOX	E-box binding factors	V$CMYC.01	Myelocytomatosis oncogene (c-myc proto-oncogene)	0.92	62	78	70	-	1	0.994	0.074	ggctccaCGTGgctccg
V$HAND	Twist subfamily of class B bHLH transcription factors	V$TWIST.01	TWIST homolog of drosophila	0.94	60	80	70	-	1	0.959	0.019	gtggctcCACGtggctccggg
V$HESF	Vertebrate homologues of enhancer of split complex	V$DEC2.01	Basic helix-loop-helix protein known as Dec2, Sharp1 or BHLHE41	0.96	63	77	70	-	1	0.968	0.008	gctccaCGTGgctcc
V$HIFF	Hypoxia inducible factor, bHLH/PAS protein family	V$ARNT.01	AhR nuclear translocator homodimers	0.89	62	78	70	+	1	0.973	0.083	cggagccaCGTGgagcc
V$EBOX	E-box binding factors	V$CMYC.01	Myelocytomatosis oncogene (c-myc proto-oncogene)	0.92	63	79	71	+	1	0.991	0.071	ggagccaCGTGgagcca
V$HESF	Vertebrate homologues of enhancer of split complex	V$DEC2.01	Basic helix-loop-helix protein known as Dec2, Sharp1 or BHLHE41	0.96	64	78	71	+	1	0.976	0.016	gagccaCGTGgagcc
V$HIFF	Hypoxia inducible factor, bHLH/PAS protein family	V$ARNT.01	AhR nuclear translocator homodimers	0.89	63	79	71	-	1	0.953	0.063	tggctccaCGTGgctcc
V$ZF08	C2H2 zinc finger transcription factors 8	V$ZNF354C.01	KRAB-zinc finger protein synten (KID3)	0.97	69	79	74	-	1	0.985	0.015	tggctCCACgt
V$CTCF	CTCF and BORIS gene family, transcriptional regulators with 1	V$CTCFL.01	CCCTC-binding factor (zinc finger protein)-like (BORIS)	0.82	69	95	82	-	0.813	0.841	0.021	gctctgggtctgaaggtGGCTccacgt
V$SPZ1	Testis-specific bHLH-Zip transcription factors	V$SPZ1.01	Spermatogenic Zip 1 transcription factor	0.94	102	112	107	+	1	0.959	0.019	aGGAGggtgac
V$GRHL	Grainyhead-like transcription factors	V$GRHL1.01	Grainyhead-like 1 (LBP32, MGR, TFCP2L2)	0.86	107	119	113	-	0.797	0.875	0.015	tctaccGGTCacc
V$ETSF	Human and murine ETS1 factors	V$FLI1.01	ETS family member FLI-1	0.81	106	126	116	+	1	0.813	0.003	gggtgaCCGGtagagcaaggc
V$KLFS	Krueppel like transcription factors	V$BKLF.01	Basic krueppel-like factor (KLF3)	0.95	124	142	133	-	1	0.956	0.006	aacggGGGTgtgcacggcc
V$MTF1	Metal induced transcription factor	V$MTF-1.01	Metal transcription factor 1, MRE	0.88	126	140	133	+	1	0.896	0.016	ccgtGCACacccccg
V$RBP2	Retinoblastoma-binding proteins with demethylase activity	V$PLU1_JARID1	Jumonji, AT rich interactive domain 1B	0.96	130	138	134	+	1	0.965	0.005	GCACacccc
V$SP1F	GC-Box factors SP1/GC	V$TIEG.01	TGFbeta-inducible early gene (TIEG) / Early growth response gene alpha (E	0.83	126	142	134	-	1	0.934	0.104	aacgGGGGtgtgcacgg
V$ZF02	C2H2 zinc finger transcription factors 2	V$ZNF219.01	Kruppel-like zinc finger protein 219	0.91	130	152	141	+	1	0.923	0.013	gcacaCCCCcgttgcagaattga
V$MYBL	Cellular and viral myb-like transcriptional regulators	V$VMYB.02	v-Myb	0.9	132	152	142	-	1	0.959	0.059	tcaattctgcAACGggggtgt
V$CEBP	Ccaat/Enhancer Binding Protein	V$CEBPE.02	CCAAT/enhancer binding protein (C/EBP), epsilon	0.97	138	152	145	-	1	0.98	0.01	tcaattctGCAAcgg
V$PARF	PAR/bZIP family	V$HLF.01	Hepatic leukemia factor	0.84	137	153	145	-	0.836	0.858	0.018	ttcaattctGCAAcggg
V$CDEF	Cell cycle regulators: Cell cycle dependent element	V$CDE.01	Cell cycle-dependent element, CDF-1 binding site (CDE/CHR tandem elem	0.87	151	163	157	-	1	0.951	0.081	gaggCGCGttttc
V$MEF2	MEF2, myocyte-specific enhancer binding factor	V$MEF2.06	Myocyte-specific enhancer factor 2	0.87	154	176	165	+	1	0.875	0.005	aacgcgcctcaAAATaaaacccc
O$VTBP	Vertebrate TATA binding protein factor	O$MTATA.01	Muscle TATA box	0.84	163	179	171	+	1	0.848	0.008	caaaaTAAAaccccgtg
V$EBOX	E-box binding factors	V$MAX.01	Max/Max dimer	0.85	168	184	176	-	1	0.856	0.006	tgaaaCACGgggtttta
V$CEBP	Ccaat/Enhancer Binding Protein	V$CEBPB.02	CCAAT/enhancer binding protein beta	0.91	177	191	184	-	1	0.995	0.085	attctTGTGaaacac
V$STAT	Signal transducer and activator of transcription	V$STAT6.01	STAT6: signal transducer and activator of transcription 6	0.84	176	194	185	-	0.793	0.882	0.042	tgcaTTCTtgtgaaacacg
V$STAT	Signal transducer and activator of transcription	V$STAT6.01	STAT6: signal transducer and activator of transcription 6	0.84	177	195	186	+	0.862	0.904	0.064	gtgtTTCAcaagaatgcac
V$HEAT	Heat shock factors	V$HSF1.01	Heat shock factor 1	0.84	175	199	187	+	1	0.847	0.007	ccgtgtttcacaAGAAtgcactaat
V$PAX2	PAX-2 binding sites	V$PAX2.01	Zebrafish PAX2 paired domain protein	0.78	177	199	188	-	1	0.831	0.051	attagtgcattcttgtgAAACac

Note: the prediction is generated by Genomatix Suit Program MatInspector (https://www.genomatix.de/online_help/help_matinspector/matinspector_help.html).

**Supplementary Table 3. DNA oligos and primers used in this study.**
For genotyping DMR CpG 3C-PCR (INS-KATAP5-6)
3CPCRp3-L
TGA ATT CCT TTG CCA TGC TCG
3CPCRp3-R
CCT GGT TAA GAC TCT AAT GAC CC
(Alternative Primers:
3CPCRp2-L
CCG TTG CAG AAT TGA AAA CGC
3CPCRp2-R
TGG TTA AGA CTC TAA TGA CCC G)
DMR(KRTAP5-6) CpG Bisulfide Sequencing primer
DMR2L1
TTT TTG TAG TTT TGG GTT TTA TTG G
DMR2R1
AAA AAA CCT ATC CTT AAA TAT AAA TCT CCA
DMR CpG Genotyping Primer
DMRL2
CCAGGCACAACTCCATCCAT
DMRR2
CCTGTCCTTGGATGTGGGTC
(Alternative Genotyping primer set:
DMRL1
CTTCCCTAGAGACCCTGGCT
DMRR1
TCCGAGCATGGCAAAGGAAT)
SNP rs2334499 Genotyping
SNP-5L
TGCAACTACCACCTCTTCCG
SNP-5R
AACAGCTGCTTCACTCTGCC
H19-Enhancer Genotyping
H19-Enhancer-5’-L
CCAAGGACAGAGGCATCAGG
H19-Enhancer-5’-R
CCCCTGTGTGTTTCATGGGA
INSP-1-3L
CTTGGTCGTCAGCACCTCTT
INSP-1-3R
GCTCAGCCCAGATGACACTA

IGF2-10L
CCACCCTCAACTCAACCCAA
IGF2-10R
CAGGCCAAAGTCCCGCTAAG
H19E-H19P 3C-PCR primers (H19P is also H19ICR)
Primer set #3 (This set does not show allele difference)
L: CACAGCTCCTTCTCGAACCT
R: TCAGGGTGAGATCCTTCTTGC
Primer set #4 (This set shows allele difference)
L: CCAGAGAGCCAAAGACCTGA
R: TCAGGGTGAGATCCTTCTTGC
H19ICR-DMR (KRTAP5-6) 3C-PCR Primer
Set #1
L: TCCACATGTCGGTGACCTCT
R: TCAGGGTGAGATCCTTCTTGC
Set #2
L: TGTGGCTTGTATGCCCAGTC
R: TCAGGGTGAGATCCTTCTTGC
H19ICR-DMR (KRTAP5-6) Long-3C-PCR Primer (contain SNPs for both loci)
Primer set #9
L:CGTGAGGCTCTGATGGTCTT
R:TGGCCTCAGGGTGAGATCC
H19ICR-CTCFDS 3C-PCR Primer
Set #3
L: CTTAGGCCCATGGACAGACC
R: GGCCTCAGGGTGAGATCCTT
KRTAP5-6-DMR-CHIP-PCR
Set #7
L: GGTCCCTGGGTGTGGATGTA
R: GCGTGGAGGTGTTTTGTGC
DMR-CHIP-PCR (alternative set)
Set #8
L: GTGGATGTAGTGACCCCGGA
R: GGCGTGGAGGTGTTTTGTG
H19 hs5 bisulfite sequencing primer
Set #1: (long product)
L: GTTGTGATGTGTGAGTTTGTATTGT
R: AATAACACAAAATTAATTATAATTATAAAA
Set #2: (short product)
L: ATTTTTAATAGTGTATTTTGGGGTGAAT
R: ACATAACACATATATATTTCTAAAAACTTC

H19ICR-IGF2 3C-PCR
H19ICR-IGF2 3C-1
GATCAGGCCAAAGTCCCGCT
H19ICR-IGF2 3C-2
GCCTCAGGGTGAGATCCTTCTT
Gel-shift DNA oligo sequence
Unmethylated DNA
F: 5’-gtagtgacccCGgagccaCGtggagccaccttcagacccagagctgaagcaggagggtgacCGg-3’
R: 5’-[Bio~dT]cCGgtcaccctcctgcttcagctctgggtctgaaggtggctccaCGtggctcCGgggtcactac-3’
Methylated DNA
F: 5’-gtagtgaccc[mC]Ggagcca[mC]Gtggagccaccttcagacccagagctgaagcaggagggtgac[mC]Gg-3’
R: 5’-[Bio~dT]c[mC]Ggtcaccctcctgcttcagctctgggtctgaaggtggctcca[mC]Gtggctc[mC]Ggggtcactac-3’
SNP rs689 Genotyping Primer
INS-7L
CTCACAACAGTGCCGGGAA
INS-7R
GGGTTGAGAGGTAGGGGAGA
CRISPR sgRNA cloning DNA oligos
CTCF site 1
1
F: CACCG GGCCGAGAAGCGCCACCTGG TGG
R: AAAC CCACCAGGTGGCGCTTCTCGGCC C
2
F: CACCG GCTGGCCGAGAAGCGCCACC TGG
R: AAAC CCAGGTGGCGCTTCTCGGCCAGC C
CTCF site 2
1
F: CACCG CAGTCCTTGAGCGCCCCCTG GGG
R: AAAC CCC CAGGGGGCGCTCAAGGACTG C
2
F: CACCG TCGGAGCTCACGGCCCCCAG GGG
R: AAAC CCCCTGGGGGCCGTGAGCTCCGA C
CTCF site 3
1
F: CACCG GGTCTGAAGGTGGCTCCACG TGG
R: AAAC CCA CGTGGAGCCACCTTCAGACC C
2
F: CACCG TGCTTCAGCTCTGGGTCTGA AGG
R: AAAC CCT TCAGACCCAGAGCTGAAGCA C
Non-target sgRNA
F: CACCG CCAGTTGCTCTGGGGGAACA
R: AAAC TGTTCCCCCAGAGCAACTGG C

Primers for cleavage indel detection assay
CTCF Site 1:
L: AAGGAATGCGCAAGTTGGTC
R: CTCCTGCCTCTGCTGTCTCT
CTCF Site 2:
L: GTTCTGTGAGGTCAACACGC
R: GTCTCTCTTCCTCCCCAAAGC
CTCF Site 3:
L: GTCACACGACTGGTGGGG
R: CAGGACGGGCTCCCGAATTT
For INS-IGF2 isoform 1 RT-PCR
INS-IGF2 RT-5L
AGCCTTTGTGAACCAACACCT
INS-IGF2 RT-5R
GACGGGCAAAGATGATCCCT
INS-IGF2 sequencing forward
GCAACTACGATATCTGTATGGATCA
Human Islet IGF2 isoform 1 RT Genotyping Primer
IGF2-1L
TCGCCGAACCAAAGTGGATTA
IGF2-1R
GAGAGTAGCCTGTTTCGGGG
Human Islet INS-IGF2 isoform 1 RT Genotyping Primer
INSIGF2-2L
CGCAGCCTTTGTGAACCAAC
INSIGF2-2R
TGATGGAAACGTCCGTGGTC
Human Islet IGF2 Sequencing Primer
ATGACACCTGGAAGCAGTCC
qRT-PCR primers:
GAPDH
L: TGCACCACCAACTGCTTAGC
R: GGCATGGACTGTGGTCATGAG
INS
L: GCAGCCTTTGTGAACCAACAC
R: CCCCGCACACTAGGTAGAGA

References:

1. Lawlor, N. *et al.* Multiomic Profiling Identifies cis-Regulatory Networks Underlying Human Pancreatic beta Cell Identity and Function. *Cell Rep* **26**, 788-801 e786, doi:10.1016/j.celrep.2018.12.083 (2019).
2. Jian, X. & Felsenfeld, G. Insulin promoter in human pancreatic beta cells contacts diabetes susceptibility loci and regulates genes affecting insulin metabolism. *Proc Natl Acad Sci U S A* **115**, E4633-E4641, doi:10.1073/pnas.1803146115 (2018).
3. Kong, A. et al. Parental origin of sequence variants associated with complex diseases. Nature 462, 868-874, doi:10.1038/nature08625 (2009).
4. Ghirlando, R. & Felsenfeld, G. CTCF: making the right connections. Genes Dev 30, 881-891, doi:10.1101/gad.277863.116 (2016).
5. Alvarez-Dominguez, J. R. *et al.* Circadian Entrainment Triggers Maturation of Human In Vitro Islets. *Cell Stem Cell* **26**, 108-122 e110, doi:10.1016/j.stem.2019.11.011 (2020).
