# Supplementary material for: Large parental differences in chromatin organization in pancreatic beta cell line explaining diabetes susceptibility effects
Source: Nat Commun. 2021 Jul 15;12:4338. doi: 10.1038/s41467-021-24635-2 (PMC8282625; doi:10.1038/s41467-021-24635-2)
Supplement: Supplementary file 2 — Reporting summary [file 41467_2021_24635_MOESM2_ESM.pdf]

nature research

Corresponding author(s): Gary Felsenfeld

Last updated by author(s): Jun 8, 2021

Reporting Summary

Nature Research wishes to improve the reproducibility of the work that we publish. This form provides structure for consistency and transparency in reporting. For further information on Nature Research policies, see our Editorial Policies and the Editorial Policy Checklist.

Statistics

For all statistical analyses, confirm that the following items are present in the figure legend, table legend, main text, or Methods section.

n/a	Confirmed	
☐	☒	The exact sample size (*n*) for each experimental group/condition, given as a discrete number and unit of measurement
☐	☒	A statement on whether measurements were taken from distinct samples or whether the same sample was measured repeatedly
☐	☒	The statistical test(s) used AND whether they are one- or two-sided<br>*Only common tests should be described solely by name; describe more complex techniques in the Methods section.*
☐	☒	A description of all covariates tested
☒	☐	A description of any assumptions or corrections, such as tests of normality and adjustment for multiple comparisons
☐	☒	A full description of the statistical parameters including central tendency (e.g. means) or other basic estimates (e.g. regression coefficient) AND variation (e.g. standard deviation) or associated estimates of uncertainty (e.g. confidence intervals)
☐	☒	For null hypothesis testing, the test statistic (e.g. *F*, *t*, *r*) with confidence intervals, effect sizes, degrees of freedom and *P* value noted<br>*Give P values as exact values whenever suitable.*
☒	☐	For Bayesian analysis, information on the choice of priors and Markov chain Monte Carlo settings
☒	☐	For hierarchical and complex designs, identification of the appropriate level for tests and full reporting of outcomes
☒	☐	Estimates of effect sizes (e.g. Cohen's *d*, Pearson's *r*), indicating how they were calculated

Our web collection on statistics for biologists contains articles on many of the points above.

Software and code

Policy information about availability of computer code

Data collection	Data collection does not require the use of any software
Data analysis	Statistical analysis of data was performed using Graphpad QuickCalcs Web site (https://www.graphpad.com/quickcalcs/) and Microsoft Excel for Microsoft 365.<br>The methylation analysis was performed with NCBI BLAST (https://blast.ncbi.nlm.nih.gov/Blast.cgi).<br>4C-seq analysis was performed with 4C-ker v1.0 (https://github.com/rr1859/R.4Cker) in R 4.0.3.

For manuscripts utilizing custom algorithms or software that are central to the research but not yet described in published literature, software must be made available to editors and reviewers. We strongly encourage code deposition in a community repository (e.g. GitHub). See the Nature Research guidelines for submitting code & software for further information.

Data

Policy information about availability of data

All manuscripts must include a data availability statement. This statement should provide the following information, where applicable:

- Accession codes, unique identifiers, or web links for publicly available datasets
- A list of figures that have associated raw data
- A description of any restrictions on data availability

All data associated with this study are available within the Article, Supplementary Information, or available from the corresponding authors upon reasonable request. Source data are provided with this paper. The 4C-seq data used in this study are available in the National Center for Biotechnology Information Gene Expression Omnibus (GEO) database under accession code GSE112346 (https://www.ncbi.nlm.nih.gov/geo/query/acc.cgi?acc=GSE112346).

April 2020

Field-specific reporting

Please select the one below that is the best fit for your research. If you are not sure, read the appropriate sections before making your selection.

☒ Life sciences ☐ Behavioural & social sciences ☐ Ecological, evolutionary & environmental sciences

For a reference copy of the document with all sections, see nature.com/documents/nr-reporting-summary-flat.pdf

Life sciences study design

All studies must disclose on these points even when the disclosure is negative.

Sample size	For colony counting experiments, we estimated that at least 10 colonies are needed to assign the contact pattern from one hetero-zygotic allele to another (homo-zygotic) allele based on Fisher's exact test. To assign the contact pattern from one hetero-zygotic allele to another heterozygotic allele, we estimated at least 8 colonies based on Fisher's exact test. Experiments for genomic contact analysis were performed with three biologically independent samples, based on standards of the field. Quantitative RT-PCR experiments were performed with five biologically independent samples, in order to apply Student's t-test for statistical analysis.
Data exclusions	No data were excluded.
Replication	Experiments for genomic contact analysis were replicated with three biologically independent samples. Quantitative RT-PCR experiments were replicated with five biologically independent samples. CTCF gel-shift chemiluminescent EMSA assay was replicated twice. Chromatin immunoprecipitation experiments were performed on three biological replicates.
Randomization	When analyzing the clones of 3C-PCR and bisulfite-PCR product, the E. coli colonies were picked up randomly. For other experiments, we analyzed all the biological samples we prepared, so sample randomization does not apply.
Blinding	The study was designed to identify the chromatin organization of a genomic region, so we analyzed all the biological replicate that are generated in our experiment. The blinding does not apply in this case.

Reporting for specific materials, systems and methods

We require information from authors about some types of materials, experimental systems and methods used in many studies. Here, indicate whether each material, system or method listed is relevant to your study. If you are not sure if a list item applies to your research, read the appropriate section before selecting a response.

Materials & experimental systems

n/a	Involved in the study	
☐	☒	Antibodies
☐	☒	Eukaryotic cell lines
☒	☐	Palaeontology and archaeology
☒	☐	Animals and other organisms
☒	☐	Human research participants
☒	☐	Clinical data
☒	☐	Dual use research of concern

Methods

n/a	Involved in the study	
☒	☐	ChIP-seq
☒	☐	Flow cytometry
☒	☐	MRI-based neuroimaging

Antibodies

Antibodies used	For the chromatin precipitation experiments, antibodies were purchased from the following sources: against CTCF (Abcam, cat. # ab70303, 1:33 dilution), against histone H3K9me3 (Abcam, cat. # ab8898, 1:50 dilution), against histone H3K27ac (Abcam, cat. # ab4729, 1:50 dilution).
Validation	The CTCF antibody (Abcam, cat. # ab70303) has been validated by western blot of immunoprecipitate from whole cell lysate of CT26.WT, 4T1, Renca, and TCMK-1 cells (https://www.abcam.com/ps/products/70/ab70303/Images/ab70303-261578-anti-ctcfantibody-<br>chip-grade-western-blot.jpg). The histone H3K9me3 antibody (Abeam, cat. # ab8898) has been valid at ed by a peptide ChIP comeptition assay using nuclear extract from mouse ES cells (https://www.abcam.com/ps/products/8/ab8898/Images/ab8898-15866-anti-histone-h3-tri-methyl-k9-antibody-chip-grade-chip.jpg). The histone H3K27ac antibody (Abcam, cat. # ab4729) has been validated by a peptide array (https://www.abcam.com/ps/products/4/ab4729/Images/ab4729-196935-anti-histone-h3-acetyl-k27-antibody-chip-grade-peptide-array.jpg) and western blot in human HeLa cell whole cell lysate (https://www.abcam.com/ps/products/4/ab4729/Images/ab4729-249105-anti-histone-h3-acetyl-k27-antibody-chip-grade-chip.jpg).

Eukaryotic cell lines

Policy information about cell lines

Cell line source(s)	EndoC-βH1 cell line was obtained via a material transfer agreement between Inserm Transfert, CNRS, Endocells, and National Institutes of Health.
Authentication	The cell line was obtained from the original developer, and was not authenticated.
Mycoplasma contamination	EndoC-βH1 cell line has been tested negative for mycoplasma contamination by PCR methods.
Commonly misidentified lines (See ICLAC register)	No commonly misidentified lines in ICLAC register were used.
